# A Deficiency in Glutamine-Fructose-6-Phosphate Transaminase 1 (Gfpt1) in Skeletal Muscle Results in Reduced Glycosylation of the Delta Subunit of the Nicotinic Acetylcholine Receptor (AChRδ)

**DOI:** 10.3390/biom14101252

**Published:** 2024-10-03

**Authors:** Stephen Henry Holland, Ricardo Carmona-Martinez, Kaela O’Connor, Daniel O’Neil, Andreas Roos, Sally Spendiff, Hanns Lochmüller

**Affiliations:** 1 Children’s Hospital of Eastern Ontario Research Institute, Ottawa, ON K1H 8L1, Canada; 2 Department of Cellular and Molecular Medicine, Faculty of Medicine, University of Ottawa, Ottawa, ON K1N 6N5, Canada; 3 Dr. Eric Poulin Center for Neuromuscular Disorders, Brain and Mind Research Institute, University of Ottawa, Ottawa, ON K1N 6N5, Canada; 4 Department of Pediatric Neurology, Center for Neuromuscular Disorders, Centre for Translational Neuro- and Behavioral Sciences, University Duisburg-Essen, 45147 Essen, Germany; 5 Medical Faculty, University Hospital Düsseldorf, Heinrich Heine University, 40225 Düsseldorf, Germany; 6 Faculty of Medicine, Medical Center, University of Freiburg, 79085 Freiburg, Germany; 7 Centro Nacional de Analisis Genomico (CNAG), 08028 Barcelona, Spain

**Keywords:** glutamine-fructose-6-phosphate transaminase 1 (Gfpt1), congenital myasthenic syndrome (CMS), neuromuscular junction (NMJ), O-GlcNAcylation, acetylcholine receptor delta subunit (AChRδ), glycosylation

## Abstract

The neuromuscular junction (NMJ) is the site where the motor neuron innervates skeletal muscle, enabling muscular contraction. Congenital myasthenic syndromes (CMS) arise when mutations in any of the approximately 35 known causative genes cause impaired neuromuscular transmission at the NMJ, resulting in fatigable muscle weakness. A subset of five of these CMS-causative genes are associated with protein glycosylation. Glutamine-fructose-6-phosphate transaminase 1 (Gfpt1) is the rate-limiting enzyme within the hexosamine biosynthetic pathway (HBP), a metabolic pathway that produces the precursors for glycosylation. We hypothesized that deficiency in Gfpt1 expression results in aberrant or reduced glycosylation, impairing the proper assembly and stability of key NMJ-associated proteins. Using both in vitro and in vivo Gfpt1-deficient models, we determined that the acetylcholine receptor delta subunit (AChRδ) has reduced expression and is hypo-glycosylated. Using laser capture microdissection, NMJs were harvested from Gfpt1 knockout mouse muscle. A lower-molecular-weight species of AChRδ was identified at the NMJ that was not detected in controls. Furthermore, Gfpt1-deficient muscle lysates showed impairment in protein O-GlcNAcylation and sialylation, suggesting that multiple glycan chains are impacted. Other key NMJ-associated proteins, in addition to AChRδ, may also be differentially glycosylated in Gfpt1-deficient muscle.

## 1. Introduction

Glycosylation, the attachment of sugar moieties to proteins, is the most common post-translational modification (PTM) associated with maintaining the structure, function, stability, and localization of proteins within the cell. Initiated within the endoplasmic reticulum (ER), these modifications are diverse in nature, ranging from the addition of a simple sugar to complex glycan chains with many branching points. Underscoring the vital role of glycans in protein homeostasis, many mutations to key genes associated with the glycosylation process are embryonically lethal [1,2]. However, some mutations within glycosylation enzymes correspond to a host of disorders called congenital disorders of glycosylation (CDGs), which typically have severe clinical manifestations impacting multiple organs and systems [3,4]. Numerous inherited muscle disorders have been associated with defects in glycosylation affecting primarily, the alpha-dystroglycan protein, including Fukuyama congenital muscular dystrophy (FCMD) [5,6], Walker–Warburg syndrome [7,8,9,10], congenital muscular dystrophy type 1C and 1D (MDC1C/D) [11,12,13,14], limb–girdle muscular dystrophy type 2I [15,16], muscle–eye–brain disease [17], Duchene’s Muscular Dystrophy [18] and GNE Myopathy [19]. 

The neuromuscular junction (NMJ) mediates the transmission of chemical signals from the pre-synaptic nerve terminal to the post-synaptic skeletal muscle, enabling muscular contraction. Congenital myasthenic syndromes (CMS) are early onset inheritable neuromuscular disorders that result from mutation to proteins that are associated with NMJ development, function, maintenance, and/or organization of the motor endplate [20]. Clinically, CMS are heterogeneous in nature but are typically characterized by fatigable muscle weakness to facial, bulbar, ocular, truncal, respiratory, limb, and/or girdle muscles [20]. A subset of five of the CMS causative genes impair protein glycosylation and results in CMS. These include glutamine-fructose-6-phosphate transaminase 1 (*GFPT1*) [21,22,23,24,25,26,27,28,29], dolichol-phosphate N-acetylglucosaminephosphotransferase-1 (*DPAGT1*) [30,31,32,33], GDP-mannose pyrophosphorylase B (*GMPPB*) [34,35], α-1,3/1,6-mannosyltransferase (*ALG2*) [36,37,38], and UDP-N-acetylglucosaminyltransferase subunit 14 (*ALG14*) [36,38]. 

Gfpt1 is the rate-limiting enzyme of the hexosamine biosynthetic pathway (HBP), a metabolic signaling pathway that produces the precursor uridine diphosphate N-acetyl-glucosamine (UDP-GlcNAc) required for N- and O-linked protein glycosylation [39,40]. The HBP metabolizes key nutrients, such as uridine triphosphate, glucose, glutamine, and acetyl-CoA, allowing for the regulation of integral processes such as cell growth [41,42], differentiation [43], stem cell differentiation [44], metabolic reprogramming [41,42], ER homeostasis [45,46,47], transcriptional control [48], and cancer pathogenesis [42]. UDP-GlcNAc is also involved in the creation of N-linked glycans through the conversion to the lipid carrier dolichol-phosphate (Dol-P) and in protein sialylation as a substrate of the UDP-GlcNAc epimerase 2 (*GNE*) enzyme [49,50]. As such, impairment in HBP activity could be associated with a deficiency in the development of a variety of glycan side chains. 

Biallelic mutations to *GFPT1* cause CMS characterized by fatigable limb–girdle muscle weakness, commonly presenting in the first years of life [22,23,24,26,28]. GFPT1-CMS muscle fibers contain tubular aggregates, which may originate from the sarcoplasmic reticulum, but their exact molecular origin remains unknown [26]. At the NMJ, GFPT1-CMS patients have reduced post-synaptic folding, reducing the efficiency of neuromuscular communication [25]. The biallelic mutations to *GFPT1* result in a decrease in GFPT1 protein levels and/or impaired enzymatic activity, which may result in reduced or aberrant glycosylation [22,24,27,51]. However, glycomics of GFPT1-CMS patient myoblasts revealed no difference in global N-linked glycan chain development, while defective N-linked glycosylation has been shown in other GFPT1 CMS patient-derived myoblasts [21,52]. This suggests the need to study muscle cells molecularly to obtain insights into the underlying pathophysiology. 

GFPT1 has been linked to protein O-GlcNAcylation, a reversible and highly dynamic modification that regulates biological function [53,54], stability, localization [48], protein–protein interactions [55], and transcriptional control [48,54]. O-GlcNAcylation involves the binding of a single GlcNAc moiety, prepared by the HBP, to serine and threonine residues on a protein via a β-configured O-glycosidic bond [56,57]. This is a reversible modification regulated by two enzymes: oligotransferase (OGT) and O-GlcNAcase (OGA) [57]. In skeletal muscle, O-GlcNAcylation remains an important regulator of the immune response [54], glucose metabolism, insulin sensitivity [53], contractile properties [55], and structural elements [55]. Impairment appears to be detrimental to various components of muscle health, and control of O-GlcNAcylation is important for various aspects of muscle function [55,58]. However, studies remain unclear whether GFPT1-deficiency impedes O-GlcNAcylation of NMJ-associated proteins. Elucidating the role of GFPT1 in protein glycosylation at the NMJ may not only reveal pathomechanisms of disease but also hint toward novel treatment strategies for CMS patients.

In this study, we hypothesize that deficient expression and enzymatic activity of GFPT1 impair protein glycosylation, resulting in the dysfunction of NMJ-associated processes. We used a previously published *Gfpt1*-CMS mouse with muscle-specific removal of GFPT1 expression, termed *Gfpt1^tm1d/tm1d^*, which recapitulates clinical features and muscle pathology of human GFPT1-CMS, including exercise-induced skeletal muscle weakness, and tubular aggregates [23,25,26]. We identified a lower-molecular-weight species of the acetylcholine receptor delta subunit (AChRδ) in the *Gfpt1*-CMS mouse. Finally, we confirmed that *Gfpt1*-deficient mice show overall impaired O-GlcNAcylation levels, resulting in impairment to downstream glycan chain development. 

## 2. Materials and Methods

### 2.1. Animal Husbandry 

Animals were housed at the University of Ottawa Animal Care and Veterinary Facility (Roger Guidon Hall, Ottawa, ON, Canada), and all breeding and experimental protocols were approved by the internal Animal Care Committee (protocols: breeding—CHEO-3089 and experimental—CHEO-3120 approved on 16 November 2018 and 2 April 2019, respectively). Mice were maintained on a 12 hr light–dark cycle, with access to food and water ad libitum. Mice were generated and genotyped as previously reported [25]. For this study, Tm1C homozygous mice (containing the two LoxP sites flanking the 7th exon of *Gfpt1* but lacking *Ckm-Cre* expression) were used as controls. *Gfpt1^tm1d/tm1d^* contains two LoxP sites flanking the 7th exon of *Gfpt1* but also contains *Ckm-Cre* to create a skeletal muscle-specific knockout of *Gfpt1* [25]. Previously, *Gfpt1^tmqd/tm1d^* mice were characterized as having knocked out Gfpt1 protein levels within cardiac and muscle tissues [25]. Also, Gfpt1 expression was found in brain and kidney, demonstrating that this model is a Gfpt1 muscle-specific knockout [25]. Mice were monitored and weighed three times per week and underwent a number of behavioral tests (see below) before tissue collection at 40 weeks.

### 2.2. Inverted Screen Test 

This was performed as described [25]. To briefly summarize, animals were suspended from an inverted wire grid, and the time to fall was recorded, with a cap of 10 min. Mice had a 30 min rest, and the test was repeated. The average time was recorded and normalized to body weight to calculate hanging impulse (g × s). This behavioral test was performed weekly.

### 2.3. Grip Strength and Fatiguability Measurements 

Repetitive grip strength measurements were conducted weekly with 8 consecutive recordings on a grip strength meter (Chatillon DFE II Grip Meter, Columbus Instruments, Columbus, OH, USA). Repetitive grip strength measurements were first conducted on the front paws. Animals then had a 30 min rest period, followed by all four paws measurements. To calculate grip strength fatigability, all measurements were normalized to body weight (g) prior to calculation:

$$Muscle\;Fatigability\;\%=\left(\frac{Average\;of\;first\;four\;measurements-Average\;of\;last\;four\;measurements}{Average\;of\;measurements}\right)$$

### 2.4. EchoMRI and Total Activity Measurements

EchoMRI body composition measurements of fat mass and lean mass were recorded weekly using the EchoMRI-700 Body Composition Analyzer (EchoMRI, Houston, TX, USA). Animals also underwent 24 h open activity monitoring using the Phenotyper™ system (Noldus Information Technology, Leesburg, VA, USA) at week 35. Total distance traveled was calculated using Phenotyper™ analysis software (EthoVision v15, Noldus Information Technology).

### 2.5. Electrophysiology and Repetitive Nerve Stimulation 

Electromyography (EMG) was performed on a *Gfpt1^tm1d/tm1d^* and Tm1C homozygous mouse to examine compound muscle action potential (CMAP) and repetitive nerve stimulation (RNS) decrement with the Cadwell Sierra Summit EMG. Mice were anesthetized with isoflurane (Baxter, #CA2L9108, Toronto, ON, Canada) prior to experimentation. To examine CMAP and RNS, recording electrodes (Cadwell US, #302353-000-100, Kennewick, WA, USA) were placed on the skin near the proximal portion of the *gastrocnemius* muscle of the hind limb, and the second electrode was placed in the *tibialis anterior*. For stimulation of the sciatic nerve, two electrodes (cathode and anode) were inserted into the proximal hind limb and subcutaneous tissue overlaying the sacrum, respectively. Submaximal CMAP responses were performed by stimulation of the sciatic nerve with a square-wave pulse of 0.1 ms duration and an intensity range of 1 to 10 mA. CMAP response was increased in stimulus intensity until the amplitude of the response was seen on the monitor. To perform RNS, a stimulation series of 8 separate twitch contractions were elicited by 10 pulses ranging between 3 Hz and 20 Hz. The stimulation series was repeated after a rest of 30 s between measurements. Decrement was then calculated between the first and the fifth stimulus. CMAP and RNS calculations were performed with the Cadwell Sierra Summit software (Version 3.1.541) using a computer with Windows 10. Mice were euthanized after the recording without regaining consciousness.

### 2.6. Immunostaining for NMJs in Whole Muscle Mounts 

Soleus muscles were fixed in 2% paraformaldehyde (PFA) for 20 min. Skeletal muscle fibers were then separated into small bundles and placed in 2% PFA overnight. The fibers were washed with phosphate-buffered saline (PBS) (Sigma, #P4417, Oakville, ON, Canada) and then placed in ice-cold Analar ethanol, then methanol for 10 min each. Fibers were then transferred to blocking/permeabilization solution (5% horse serum, 5% bovine serum albumin, 1% Triton X-100 (Sigma, #X100, Oakville, ON, Canada) in PBS) and incubated for 4 h. The muscle fibers were then incubated with anti-SV2 (DSHB, 1:100, University of Iowa, Iowa City, IA, USA) and neurofilament (DSHB (2H3), 1:100, University of Iowa, Iowa City, IA, USA) in 5% horse serum, 5% BSA, for 24 h.

Fibers were then washed with PBS for 4 h and incubated with secondary antibodies: alpha-bungarotoxin-488 (Invitrogen, #B13422, 1:200, Burlington, ON, Canada) and Alexa Fluor anti-mouse-568 (Life Technologies, #A11031, 1:250, Toronto, ON, Canada) overnight. Following this incubation, the muscle fibers were washed with PBS for 4 h and mounted on glass microscope slides using Vectashield^®^ Antifade Mounting Medium (Vector Laboratories, #H-1000-10, Newark, CA, USA). Prior to mounting the tissue, several layers of electrical tape were used to create a well to hold the tissue in place. 

NMJ z-stacks were captured using a confocal microscope (Olympus Fluoview FV-1000, Olympus, Richmond Hill, ON, Canada) at 63× magnification and converted to maximum-intensity projections. Image acquisition was performed sequentially with constant microscope settings.

### 2.7. Labeling of NMJs for Laser Capture Microdissection

Gastrocnemius muscles were isolated from mice, embedded in O.C.T compound (Fisher Healthcare Tissue-Plus™, FisherScientific, #23-730-571, Ottawa, ON, Canada), and stored at −80 °C until sectioning. Gastrocnemius muscles were cryosectioned (Leica Biosystems, CM1860, Concord, ON, Canada) longitudinally at 10 μm, placed on Superfrost™ Plus glass microscope slides (FisherScientific, #12-55015), and air-dried for 1 h at room temperature. Sections were fixed with ice-cold acetone for 15 min at 4 °C and washed with PBS. Next, sections were incubated with permeabilization solution (PBS, 0.1% Triton X-100) for 15 min at room temperature, then washed with PBS. Then, muscle sections were incubated with blocking buffer (1% BSA, 10% normal goat serum (NGS) in PBS) for 30 min at room temperature. Finally, the muscle sections were incubated with α-bungarotoxin-594 (Fisher Scientific, #B13423, 1:100, Ottawa, ON, Canada) for 2 h and washed with PBS. Muscle sections were stored at 4 °C and allowed to air dry for 1 h before laser capture microdissection.

### 2.8. Laser Capture of NMJs 

NMJs were identified using a PALM MicroBeam laser capture microdissection microscope at 20× magnification (Carl Zeiss Laser Microdissection, PALMRobo 48 FTSS355-Laser System, Dorval, QC, Canada). The laser speed during cutting was set at 5%, while the laser energy was set to 70 Hz, with a Delta of 60 Hz. 

NMJs and neighboring laser-captured muscle were collected in separate Zeiss AdhesiveCap opaque 500 Eppendorf tubes (Zeiss, #415190-9201-000, Dorval, QC, Canada). Proteins were extracted by incubating the samples with RIPA buffer (containing phosphatase and protease inhibitors) for 1 h on ice, followed by centrifugation (Sorvall Legend Micro 21 R, ThermoScientific™, Ottawa, ON, Canada) at 15,000× *g* for 20 min. The supernatants were combined with Laemmli buffer and boiled at 100 °C for 5 min, and proteins were resolved with SDS-PAGE.

### 2.9. Cell Culture 

C2C12 cells (ATCC, #CRL-1722, Burlington, ON, Canada) were cultured in Dulbecco’s Modified Eagle Medium (DMEM) high-glucose media supplemented with 10% fetal bovine serum (FBS), 1% glutamine, and 1% penicillin/streptomycin. When myoblasts reached 95% confluence, the cells were washed with PBS, and the growth medium was replaced with differentiation medium (DMEM high glucose, 2% horse serum, and 1% penicillin/streptomycin) to promote myoblast fusion into myotubes. After five days, C2C12 myotubes were imaged with phase-contrast light microscopy (EVOS Cell Imaging Systems, Thermofisher, Ottawa, ON, Canada) confirm the presence of myotubes (elongated cells with more than one nuclei). All cell experiments were maintained in a humidified environment at 37 °C with 5% CO_2_.

The GFPT1/Gfpt1 inhibitor, 6-diazo-5-oxo-l-norleucine (DON) (500 μM, Sigma, #D2141, Oakville, ON, Canada), and the Oga inhibitor Thiamet-G (TG) (5 μM, Cayman, #13237, Ann Arbor, MI, USA) were administered for 48 h. 

### 2.10. Transducing C2C12 Cells with Lentiviruses Expressing shGfpt1 Transgenes

C2C12 cells were transduced with a lentivirus encoding a tetracycline repressor (TET-R) transgene (VectorBuilder, pLV(EXP)-CMV>tTS/rtTA/Hygro, Vector ID: VB161122-1052kwm, Chicago, IL, USA) at multiplicities of infection (MOIs) ranging from 1 to 500. Selective pressures were applied by adding 500 μg/mL of hygromycin B (Sigma, #400053, Oakville, ON, Canada) to generate a polyclonal stable cell line. *TET-R* expression was confirmed through RT-qPCR. C2C12 polyclonal TET-R positive cells were seeded to generate single-cell monoclonal colonies expressing TET-R. 

Monoclonal TET-R positive C2C12 cells were then infected with lentiviruses containing the transgenes for either a scramble sequence (Vector ID: VB200625-1130ssr), shGfpt1 #1 (Vector ID: VB200624-1008wvj), or shGfpt1 #2 (Vector ID: VB200625-1131yeg), each containing a tetracycline promoter and an eGFP reporter. C2C12 cells were infected with these viruses at varying Multiplicity of Infections (MOIs), and 5 μg/mL of puromycin (Life Technologies, #A1113803, Toronto, ON, Canada) was applied as a selective pressure to establish a stable polyclonal expressing cell line. 

To induce the expression of the transgenes, 2 μg/mL of doxycycline (Sigma, #D9891) was added. After 48 h, eGFP expression in lentiviral-infected C2C12 cells was documented using an EVOS cell imager. Polyclonal colonies were individually assessed to confirm the knockdown of Gfpt1 and subsequent activation of eGFP signal before monoclonal colony expansion. 

### 2.11. Fluorescence-Activated Cell Sorting (FACS) of Gfpt1-Deficient C2C12 Myoblasts

Doxycycline-inducible scramble, shGfpt1 #1, and shGfpt1 #2 C2C12 myoblasts were seeded onto 15 cm plates and treated with 2 μg/mL of doxycycline for 72 h. eGFP expression was assessed via an EVOS cell imager prior to FACS (Sony Biotechnologies SH800 Cell Sorter, San Jose, CA, USA). C2C12 cells were trypsinized, centrifuged, and prepared to a concentration of 1.0 × 10^6^ cells/mL. Dead cells were identified with 1 μg/mL of propidium iodide (PI) (STEMCELL Technologies, #75002, Vancouver, BC, Canada). 

Cells were then sorted into the following groups: eGFP−/PI−, eGFP+/PI−, eGFP−/PI+, and eGFP+/PI+. Subsequently, eGFP+/PI− and eGFP−/PI+ populations were used to establish gates for removing any autofluorescence signal. Finally, a final population of eGFP+/PI+ cells was isolated and plated into a 6-well plate for expansion. 

### 2.12. Gfpt1 Enzymatic Activity Assay

The enzymatic activity of Gfpt1 was measured using the glutamate dehydrogenase method, as previously reported [22,51]. C2C12 cells were treated with a range of doses (between 0 μM and 500 μM) of Gfpt1 inhibitor DON for 48 h. Additionally, scramble, shGfpt1 #1, and shGfpt1 #2 C2C12 myotubes were assessed for Gfpt1 enzymatic activity after 72 h of induction with 2 μg/mL of doxycycline. The change in absorbance is monitored at a wavelength of 370 nm (OD370) with a spectrophotometer microplate reader (BioTek Synergy HTX Multimode Reader, Santa Clara, CA, USA) using Agilent Biotek Gen5 software v2.06. The enzymatic activity of each condition was normalized to the Gfpt1 protein levels determined by Western blot. All measurements were performed in triplicate.

### 2.13. Real-Time qPCR (RT-qPCR)

RNA collection for in vitro experiments was performed using the RNeasy™ Micro mini kit (Qiagen, #74134, Toronto, ON, Canada). Skeletal muscle RNA extraction was performed with the RNeasy™ Fibrous Micro mini kit (Qiagen, #74704) and quantified by nanodrop. RNA was converted to cDNA with the All-In-One 5x RT MasterMix (ABM, #G592, Richmond, BC, Canada). qPCR was performed using the PowerUp™ SYBR™ Green Master Mix (FisherSci, #A25741, Ottawa, ON, Canada) with the QuantStudio™ Studio 6 Flex Real-Time PCR System (Thermofisher, Ottawa, ON, Canada) (Supplementary Materials Table S1). 

### 2.14. Tissue/Cell Lysis and Western Blotting

Cell lysates and skeletal muscle homogenates were prepared with RIPA buffer supplemented with protease (Roche, #04693116001, Mississauga, ON, Canada) and phosphatase (Roche, #PHOSS-RO) inhibitors, as well as Thiamet-G (Cayman, #13237) to prevent the loss of O-GlcNAc modification. Samples were centrifuged at 15,000× *g* for 10 min at 4 °C, and the supernatant was collected. For muscle homogenates, tissue was combined with lysis buffer and a metal bead in 2 mL SafeLock™ Eppendorf tubes (Eppendorf, #022363352, Mississauga, ON, Canada). Samples were then subjected to homogenization using a TissueLyser II (Qiagen, Toronto, ON, Canada) at 30 Hz for 90 s, followed by an incubation on ice for 60 s. This process was repeated 3 times. After homogenization, samples were left at 4 °C for 4 h on a rotating platform and then centrifuged at 15,000× *g* for 15 min at 4 °C. Total protein concentration was determined using a DC Protein Assay with BSA as a standard. Proteins were resolved by SDS-PAGE and transferred to PVDF membranes using a semi-dry BioRad™ Trans-Blot Turbo (#1704150EDU, Mississauga, ON, Canada transfer system. For assessment of poly-sialyation measurements, a wet-transfer was performed overnight at 40 V. 

Membranes were blocked with Intercept TBS blocking buffer (Li-Cor, #927-6000, Lincoln, NE, USA) and incubated with primary antibodies in blocking solution overnight at 4 °C. After washing with TBS + 1% Tween-20, membranes were incubated with IR Dye^®^800CW Donkey anti-Mouse IgG (Li-Cor, #926-32212, 1:5000) and IR Dye^®^680RD Goat anti-Rabbit IgG (Li-Cor, #926-68071, 1:5000) for 1 h at room temperature. Proteins were visualized using a Licor Odyssey, and densitometry was performed using Image Studio™ (Licor, version 6.0).

### 2.15. PNGase F and α2-3, 6, 8 Neuraminidase Reactions 

To examine the glycosylation status of the AChRδ subunit, 20 μg of tissue lysates were combined with either PNGaseF (New England Biolabs, #P0704S, Whitby, ON, Canada) or α2-3, 6, 8 Neuraminidase (New England Biolabs, #P0720S) according to manufacturer’s instructions. AChRδ protein levels and molecular weight were assessed via Western blot. 

### 2.16. Antibodies

The following antibodies were used for Western blot experiments: Gfpt1 (ProteinTech, #14132-1-AP, 1:500, Rosemont, IL, USA), Gapdh (14C10) (Cell Signalling, #2118, 1:2000, Danvers, MA, USA), O-GlcNAc (RL2) (Novus Biologicals, #NB300-524, 1:1000, Toronto, ON, Canada), Ogt (ProteinTech, #66823-1-Ig, 1:1000, Rosemont, IL, USA), Desmin (ProteinTech, #16520-1-AP, 1:1000, Rosemont, IL, USA), AChRα (ProteinTech, #10613-1-AP, 1:1000, Rosemont, IL, USA), AChRδ (C-4) (Santa Cruz, #sc-390896, 1:1000, Dallas, TX, USA), PolySia 735 (gift from Dr. Rita Gerardy-Schahn, Hannover, Germany, 1:1000), Vinculin (7F9) (Santa Cruz, #sc-73614, 1:2000, Dallas, TX, USA), Lrp4 (extracellular) (NeuroMab N207/27, #75-221, UC Davis/NIH NeuroMab Facility, Davis, CA, USA), biotinylated-succinylated wheat germ agglutinin (sWGA) (Vector Laboratories, #B-1025S-5, 1:1000, Newark, CA, USA), and IRDye^®^ 800CW Streptavidin (Li-Cor, #926-32230, 1:5000). For NMJ staining from whole muscle mounts, SV2 antibody (DSHB, #, 1:250, The University of Iowa, Department of Biology, Iowa City, IA, USA), and neurofilament M were used (DSHB, 2H3, 1:250). 

### 2.17. Statistics

Data were analyzed with GraphPad Prism v 10.1.2. Data were checked for normality using the D’Agostino-Pearson test, and the appropriate statistical test was applied as described in the figure legends. Any outlier was identified using the robust regression and outlier remover method (ROUT). 

Statistical significance was determined by using unpaired two-tailed Student’s *t*-tests or analysis of variance (ANOVA). If multiple comparisons were performed, a Tukey’s post hoc test was performed. Results are given as mean ± S.D, and *p* < 0.05 was considered significant. 

## 3. Results

### 3.1. Skeletal Muscle Knockout of Gfpt1 (Gfpt1^tm1d/tm1d^) in Mice Hasprogressive Muscle Fatigability with Impaired NMJ Morphology

**Figure 1:**
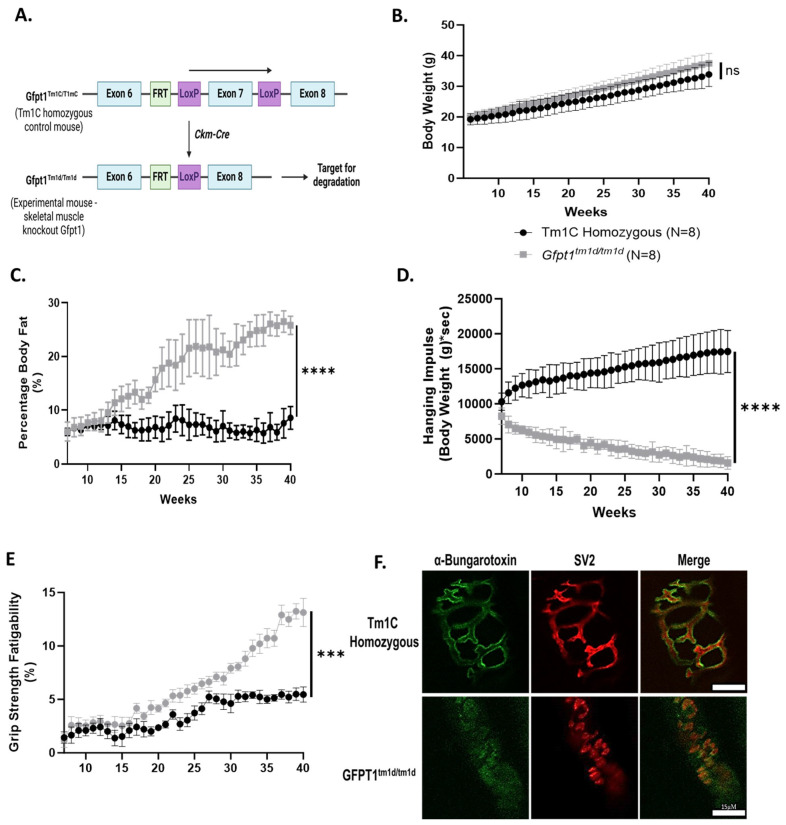
*Gfpt1^tm1d/tm1d^* mice exhibit progressive fatigable muscle weakness with disrupted NMJ morphology. (**A**) Our previously published *Gftp1^tm1d/tm1d^* mouse model was developed with Cre/LoxP technology. This model contains two LoxP sites, which flank the seventh exon of Gfpt1 (termed *Gftp1^tm1c/tm1c^*). Gfpt1^tm1c/tm1c^ mice are crossed with the Ckm-Cre expressing mouse to produce a heterozygous mouse containing Ckm-Cre and the LoxP flanking Gfpt1, termed *Gfpt1^Cre-Het^*. The *Gfpt1^Cre-Het^* mice were crossed with *Gfpt1^tm1c/tm1c^* mice to produce the *Gfpt1^tm1d/tm1d^* mouse line. The *Gfpt1^tm1d/tm1d^* mouse model expresses a skeletal muscle-specific truncation of Gfpt1, which is targeted for degradation. (**B**) Mice were weighed 3 times per week over 40 weeks; *Gfpt1^tm1d/tm1d^* mice (grey line) showed no significant difference in body weight when compared to Tm1C homozygous control mice (black line). (**C**) There was a significant increase in body fat percentage in *Gfpt1^tm1d/tm1d^* mice over the same period (EchoMRI). (**D**) *Gfpt1^tm1d/tm1d^* mice have a progressive reduction in hanging impulse when compared to Tm1C homozygous control mice. (**E**) Repetitive grip strength measurements demonstrated that *Gfpt1^tm1d/tm1d^* mice have increased muscle fatigability compared to Tm1C homozygous control mice. (**F**) NMJs from soleus muscle were labeled with anti-synaptic vesicle 2 to label pre-synaptic nerve terminals (red) and α-bungarotoxin to visualize AChR clusters (green). All graphs show mean ± SD, and statistical significance was determined via a two-way ANOVA. *n* = 8 for both Tm1C homozygous control mice and Gfpt1^tm1d/tm1d^ mice. *** *p*-value < 0.001, **** *p*-value < 0.0001, ns: not significant.

We used the previously published Gfpt1 skeletal muscle-specific knockout mouse model termed *Gfpt1^tm1d/tm1d^* for these investigations [25]. These mice express two LoxP sites flanking the seventh exon of Gfpt1, which also contains Cre recombinase controlled by the creatine kinase (Ckm) promoter (Figure 1A). This causes truncation to occur within Gfpt1, targeting it for degradation. These mice lack *Gfpt1* gene expression and thus the corresponding protein in muscle (Supplementary Figure S1A,B). The previous characterization of these mice revealed modest behavioral and phenotypic changes as well as NMJ abnormalities up to 26 weeks of age [25]. In this study, we used 40-week-old mice to help identify the phenotypic and molecular changes, anticipating these would be progressive and more pronounced in older animals (Supplementary Figure S1C). 

Over 40 weeks, *Gfpt1^tm1d/tm1d^* mice did not exhibit significant differences in body weight compared to Tm1C homozygous control animals (Figure 1B). However, they did have a significant increase in body fat percentage, reflecting a decrease in lean body mass (Figure 1C). *Gfpt1^tm1d/tm1d^* mice also demonstrated a progressive impairment in the latency to fall (Figure 1D and Supplementary Figure S1D) and no significant change in maximal grip strength, but an increase in grip strength fatigability was observed (Figure 1E and Supplementary Figure S1E–G). Also, a significant decrease in total mouse activity was noted (Supplementary Figure S1H). Next, we performed EMG recordings in an older (54-week-old) *Gfpt1^tm1d/tm1d^* and Tm1C homozygous mouse. The *Gfpt1^tm1d/tm1d^* mouse had a shorter amplitude in sub-maximal CMAP measurements (14.8 mV to 36.1 mV) (Supplementary Figure S2A). The sub-maximal CMAP was used for RNS recordings at low frequency (3 Hz) and high frequency (20 Hz). We found that the *Gfpt1^tm1d/tm1d^* experienced a decrement at low-frequency (~12% at 3 Hz) and high-frequency (~25% at 20 Hz) RNS exceeding 10%, while minimal decrement was measured within the Tm1C homozygous control mouse (Supplementary Figure S2B,C). A CMAP decrement of 10% is commonly used as a threshold for diagnosing muscular fatigue in CMS patients [20,59]. Finally, examination of the NMJs revealed that *Gfpt1^tm1d/tm1d^* mouse skeletal muscle had smaller and less complex NMJs when compared to Tm1C homozygous control animals (Figure 1F). These results confirm our previous study demonstrating that *Gfpt1^tm1d/tm1d^* mice exhibit increased muscle fatigability, reduced grip strength, and impaired NMJ morphology. The maintenance of body weight with an increasing body fat percentage suggests muscle loss may be occurring, explaining in part the changes in fatigue, strength, and failure of neuromuscular transmission. 

### 3.2. Gfpt1^tm1d/t1md^ Mice Exhibit Hypo-Glycosylated AChRδ Subunits 

**Figure 2:**
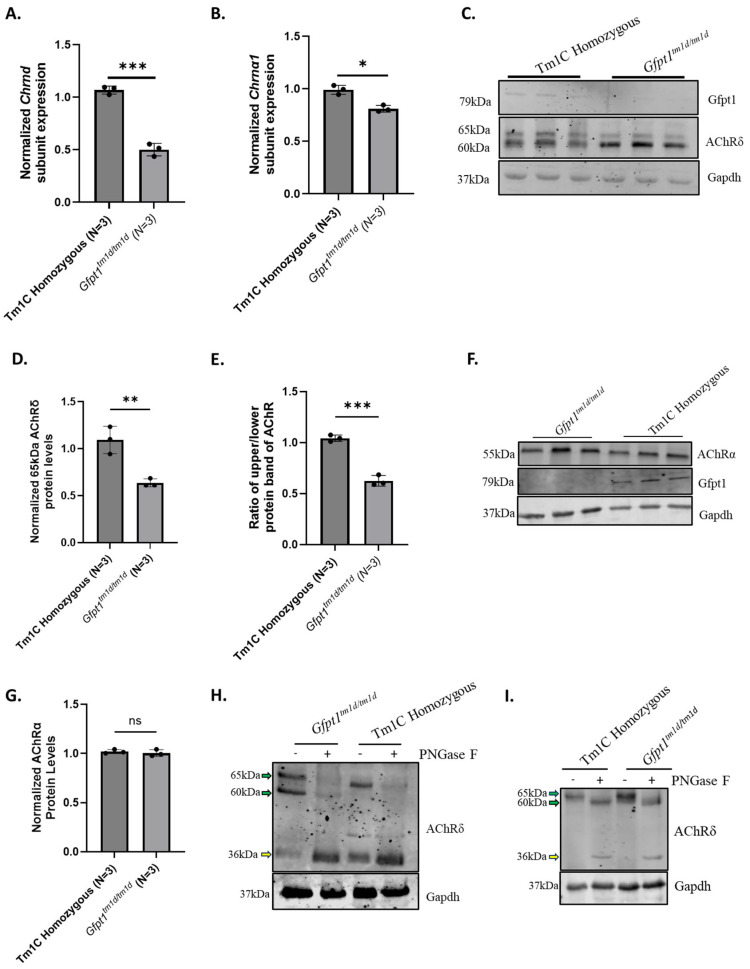
*Gfpt1^tm1d/tm1d^* mice exhibit impaired *Chrnd* gene and AChRδ protein expression with the presence of an altered molecular weight banding pattern. (**A**) *Chrnd* and (**B**) *Chrna1* gene expression are reduced in quadriceps isolated from 40-week-old *Gfpt1^tm1d/tm1d^* mice normalized to reference gene expression. (**C**) A low-molecular-weight species (60 kDa) can be detected in AChRδ in *Gfpt1^tm1d/tm1d^* skeletal muscle lysates on Western blot. (**D**) AChRδ protein levels are reduced in *Gfpt1^tm1d/tm1d^* skeletal muscle compared to Tm1C homozygous control mice, normalized to Gapdh loading control. (**E**) *Gfpt1^tm1d/tm1d^* mice exhibited a reduction in the ratio of the high-molecular-weight species and the low-molecular-weight species of AChRδ upon comparison to Tm1C homozygous control mice. (**F**) Provided the change in AChRδ, we examined additional AChR subunit protein levels. There was no discernable change in molecular weight with AChRα. (**G**) There was no significant change in AChRα protein levels within *Gfpt1^tm1d/tm1d^* quadricep. (**H**) A Western blot showing quadriceps and (**I**) gastrocnemius muscle lysates from *Gfpt1^tm1d/tm1d^* and Tm1C homozygous control following treatment with PNGase F. PNGase F treatment resulted in the migration of the 65 kDa and 60 kDa species of AChRδ species, suggesting that the lower molecular weight is hypo-glycosylated. The molecular weight species of AChRδ are labeled with 65kDa and 60kDa in green. A lower 36kDa species is labelled with yellow arrows. For in vivo experiments assessing protein levels and RNA expression, three mice were assessed (*n* = 3). Original images can be found in Supplementary File S1. Graphs show mean ± SD; statistical significance was determined by Student’s *t*-Test. * *p*-value < 0.05, ** *p*-value < 0.01, *** *p*-value < 0.001, ns: not significant.

A previous study indicated that a reduction in Gfpt1 protein levels resulted in a decrease in AChR subunit expression within TE671/DB40 cells [28]. Therefore, we investigated the expression of AChR subunits within skeletal muscle isolated from *Gfpt1^tm1d/tm1d^* mice. *Gfpt1^tm1d/tm1d^* skeletal muscle exhibited a significant reduction in Chrnd and Chrna1 RNA (Figure 2A,B) and AChRδ protein levels in the quadriceps (Figure 2C,D). Furthermore, AChRδ appeared as two bands on the Western blot of both Tm1C homozygous control and *Gfpt1^tm1d/tm1d^* skeletal muscle, a high-molecular-weight species at ~65 kDa and a low-molecular-weight species at ~60 kDa (Figure 2C). The low-molecular-weight species of AChRδ was more prominent in Gfpt1^tm1d/tm1d^ quadriceps muscle than control animals (Figure 2C,E). This effect was also observed in the gastrocnemius muscle (Supplementary Figure S3A–C). No drop in protein levels or a second molecular weight species of the AChRα subunit was visible in Gfpt1^tm1d/tm1d^ quadriceps muscle (Figure 2F,G). Also, we assessed Lrp4 protein levels in whole muscle lysates. We established that there was also no drop in protein levels or a second molecular weight species visible within *Gfpt1^tm1d/tm1d^* quadriceps (Supplementary Figure S3D,E). The prominence of the lower molecular weight of AChRδ in *Gfpt1^tm1d/tm1d^* prompted us to determine its origin. 

The AChRδ subunit is a tightly regulated glycoprotein with several previously reported sites for glycosylation [60,61]. We used the N-linked glycanase, PNGaseF, which cleaves the innermost GlcNAc modification of complex oligosaccharides bound to asparagine residues, to determine if the lower AChRδ molecular weight species is differentially glycosylated in *Gfpt1^tm1d/tm1d^* muscle. In both *Gfpt1^tm1d/tm1d^* muscle and Tm1C homozygous control muscle lysates, the high-molecular-weight species disappeared after PNGase F treatment, to its unglycosylated core species (Figure 2H,I). Additionally, the low-molecular-weight species of AChRδ found in *Gfpt1^tm1d/tm1d^* skeletal muscle also disappeared following treatment with PNGase F (Figure 2H,I). This indicates that both the molecular weight species of AChRδ are glycoproteins and that the low-molecular-weight species shows an altered, potentially immature, or aberrant glycosylation profile. 

UDP-GlcNAc, the product of the HBP, can be used for terminal N-linked glycan chain substrates such as sialyation. Sialyation is a terminal glycan chain PTM, which has been implicated in various disorders such as GNE Myopathy and sarcopenia. We wanted to investigate whether protein sialylation is deficient in Gfpt1-depleted mouse muscle. While poly-sialylation was significantly reduced in *Gfpt1^tm1d/tm1d^* quadriceps (Supplementary Figure S3F,G), there were no changes in sialyation of the AChRδ subunit and Desmin (Supplementary Figure S3H). This indicated that AChRδ is siaylated and that modification did not account for a molecular weight shift. Taken together, these results suggest that AChRδ has multiple glycoproteins present, which are differentially expressed in *Gfpt1^tm1d/tm1d^* skeletal muscle, and that this low-molecular-weight species may be an immature glycoprotein.

### 3.3. Gfpt1-Deficient C2C12 Cells Exhibit Reduced AChRδ Protein Levels with a Low-Molecular-Weight Species

**Figure 3:**
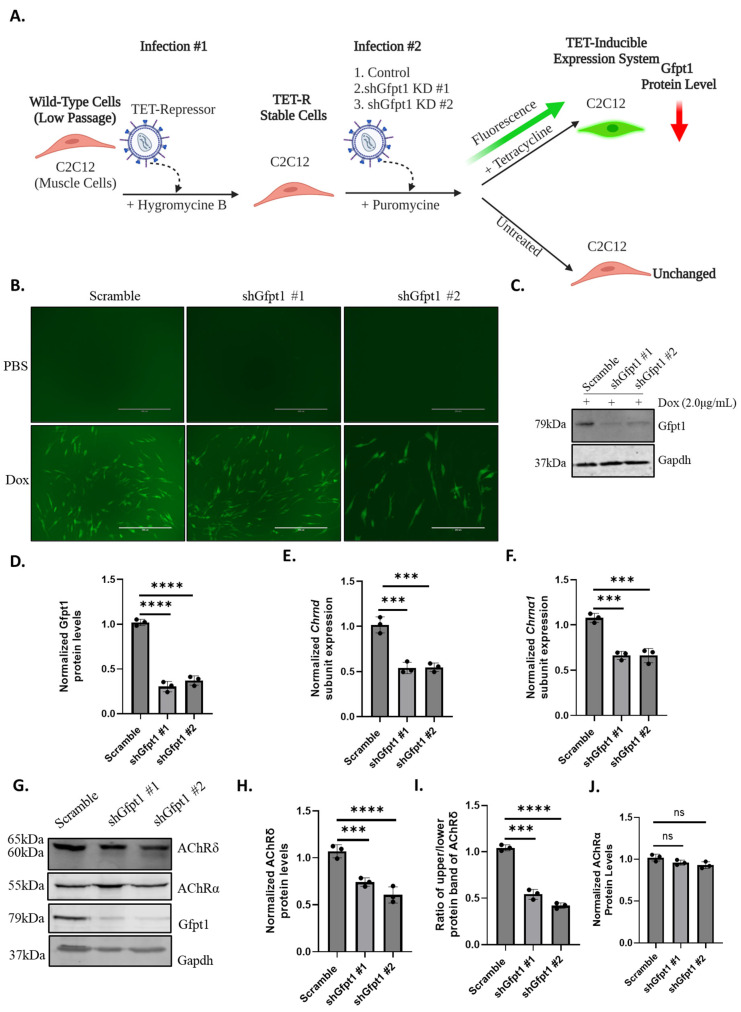
Gfpt1 deficient C2C12 myotubes exhibit impaired AChRδ protein levels, with the presence of a low-molecular-weight species. (**A**) Gfpt1-deficient C2C12 myoblasts were created using a tetracycline-inducible lentiviral system. A lentivirus encoding a tetracycline repressor (TET-R) was used to infect C2C12 cells under the selection of hygromycin. Next, a scramble, shGfpt1 #1 and shGfpt1 #2 expressing transgene, was infected into Tet-R positive C2C12 cells. Cells infected with the scramble and Gfpt1-deficient transgenes were selected with puromycin. (**B**) Exposure to doxycycline activates an eGFP reporter in scramble, shGfpt1 #1 and shGfpt1 #2 stable C2C12 cells. Scale bar is 200 μm. Gfpt1 protein levels were found in Gfpt1-deficient C2C12 myoblasts upon activation with doxycycline. (**C**,**D**) A reduction in Gfpt1 protein levels after treatment with doxycycline in Gfpt1-deficient C2C12 myoblasts was observed. (**E**) Gfpt1-deficiency in C2C12 myotubes impairs the expression of Chrnd and (**F**) Chrna1. (**G**) Gfpt1-depleted myotubes express a lower-molecular-weight 60 kDa species of AChRδ that was not present in scramble control conditions. (**H**) A reduction in AChRδ protein levels in Gfpt1-depleted C2C12 myotubes was observed. (**I**) Densitometry was used to show a reduction in the ratio of the upper molecular weight of AChRδ and the low-molecular-weight species of AChRδ in C2C12 myotubes. (**J**) There was no significant change in AChRα protein levels between scramble and Gfpt1-deficient myotubes. For in vitro experiments, protein levels and RNA expression were analyzed by three separate experiments (*n* = 3). Original images can be found in Supplementary File S1. Graphs are presented as mean ± SD, and statistical significance was determined by one-way ANOVA. ns *p*-value > 0.05, *** *p*-value< 0.001, **** *p*-value < 0.0001, ns: not significant.

To further investigate the impact of Gfpt1 deficiency on cellular processes, we established a Gfpt1-depleted C2C12 cell line (Figure 3A) using a lentiviral system to infect myoblasts with a tetracycline-inducible scramble, shGfpt1 #1 or shGfpt1 #2 transgene (Figure 3B, Supplementary Figures S4A–F). These myoblasts had a ~70% and ~63% reduction in Gfpt1 protein levels with shGtpt1 #1 and 2, respectively, when compared to scramble (Figure 3C,D). 

Next, we wanted to determine if our Gfpt1-deficient C2C12 myotubes had reduced AChR subunit expression. Following activation with doxycycline, both *Chrnd* and *Chrna1* expression levels were reduced by nearly ~50% in Gfpt1-depleted myotubes (Figure 3E,F). In addition, a decrease was also observed in the protein levels of AChRδ. (Figure 3G,H). Similarly to *Gfpt1^tm1d/tm1d^* skeletal muscle, AChRδ expressed two molecular weight species in Gfpt1-depleted myotubes (Figure 3G). The expression of the low-molecular-weight species in Gfpt1-depleted C2C12 myotubes led to a reduced ratio in AChRδ protein levels of the upper- to lower-molecular-weight band (Figure 3I). In addition, as observed in *Gfpt1^tm1d/tm1d^* skeletal muscle, there was no change in AChRα protein levels in Gfpt1-deficient C2C12 myotubes (Figure 3G,J). Taken together, these results support our findings in *Gfpt1^tm1d/tm1d^* mice, indicating that impairment to Gfpt1 causes altered glycosylation of AChRδ, resulting in the increased expression of a low-molecular-weight species. 

### 3.4. NMJ-Specific Expression of the Lower-Molecular-Weight AChRδ Subunit

Whole-muscle lysates have diluted levels of NMJ-associated proteins in large-scale proteomic analysis, and these analyses do not reveal whether the NMJ proteins are correctly localized [62]. Therefore, we utilized laser capture microdissection (LCM) to isolate NMJs from cryo-sectioned skeletal muscle to assess NMJ-associated protein expression. We first validated this approach in gastrocnemius muscle isolated from 25-week-old C57Bl/6N control mice (Supplementary Figure S5). NMJ-associated proteins AChRα, AChRδ, SV2, and MuSK were only identified in the NMJ isolate but not in muscle tissue taken from bungarotoxin-negative areas (Supplementary Figure S5D). The bungarotoxin-negative areas served as our material with non-NMJs detected and were excised after each NMJ was collected. Additionally, both Desmin and Gapdh were detected in each isolate, suggesting that this enrichment technique successfully isolated NMJ-associated proteins.

**Figure 4:**
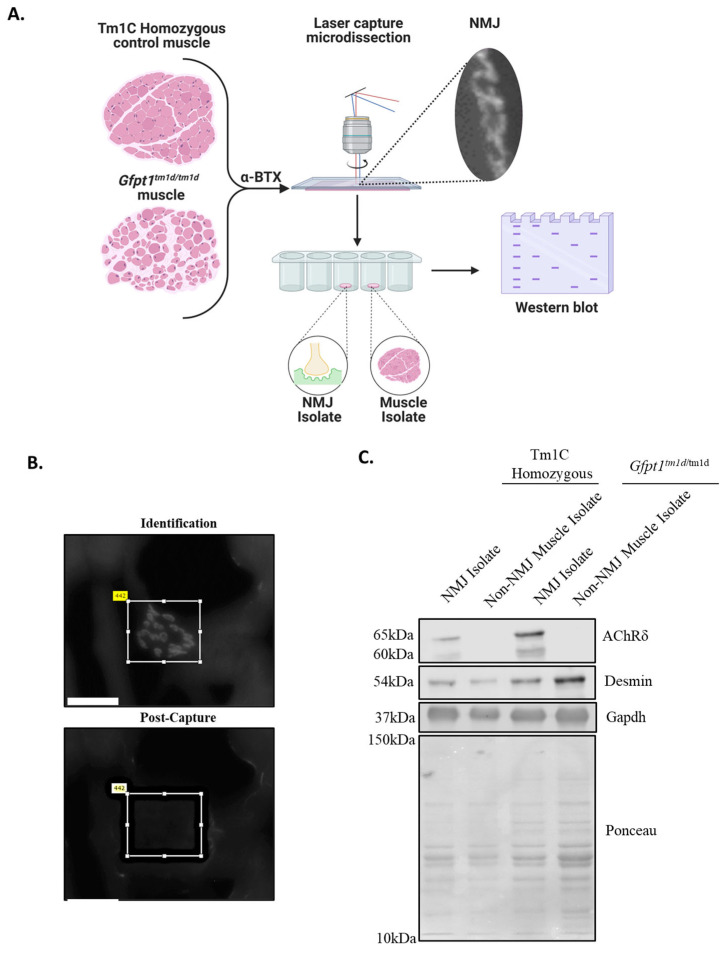
The low-molecular-weight species is detected in the NMJs isolated from *Gfpt1^tm1d/tm1d^* muscle. (**A**) Laser microdissection was used to enrich NMJs in *Gfpt1^tm1d/tm1d^* and Tm1C homozygous control mice. Gastrocnemius muscles were longitudinally sectioned and stained for AChR clusters. (**B**) An image at 40× magnification of AChR clusters before and after isolation. Scale bar: 500 μm. (**C**) A Western blot of NMJ and muscle isolates from *Gfpt1^tm1d/tm1d^* and Tm1C homozygous control mice for the detection of AChRδ, Desmin, and Gapdh. *Gfpt1^tm1d/tm1d^* NMJ isolates express an upper- and low-molecular-weight species for the AChRδ subunit, which was not observed in Tm1c homozygous control NMJ isolate. AChRδ was used to assess for NMJ-specific protein detection, while desmin and Gapdh were used to examine expression in both NMJ and muscle isolates to ensure sample purity. Original image can be found in Supplementary File S1.

To examine if both molecular weight species of AChRδ were found at the NMJs of Gfpt1^tm1d/tm1d^ mice, the muscle was sectioned longitudinally and stained for AChR clusters (Figure 4A). Approximately 1000 NMJs were isolated from *Gfpt1^tm1d/tm1d^* and Tm1C homozygous control mice (Figure 4B, Supplementary Figure S6A,B). Slightly more NMJs were collected from *Gfpt1^tm1d/tm1d^* mice, as previous studies had reported that *Gfpt1^tm1d/tm1d^* mice NMJs were smaller compared to the Tm1C homozygous control (Supplementary Figure S6C). NMJ isolates expressed the AChRδ protein, which was absent in skeletal muscle (Figure 4C). The *Gfpt1^tm1d/tm1d^* NMJ isolate sample expressed both the upper- and low-molecular-weight species of AChRδ (Figure 4C). The low-molecular-weight species were more abundant within the *Gfpt1^tm1d/tm1d^* NMJ isolate compared to the Tm1C homozygous control NMJs. Taken together, these results indicate that the low-molecular-weight species localizes to the NMJ within all mice but is present in greater abundance in *Gfpt1^tm1d/tm1d^* NMJs, which may alter the function of the AChR. 

### 3.5. Gfpt1-Depleted Models Have Impaired O-GlcNAcylation

**Figure 5:**
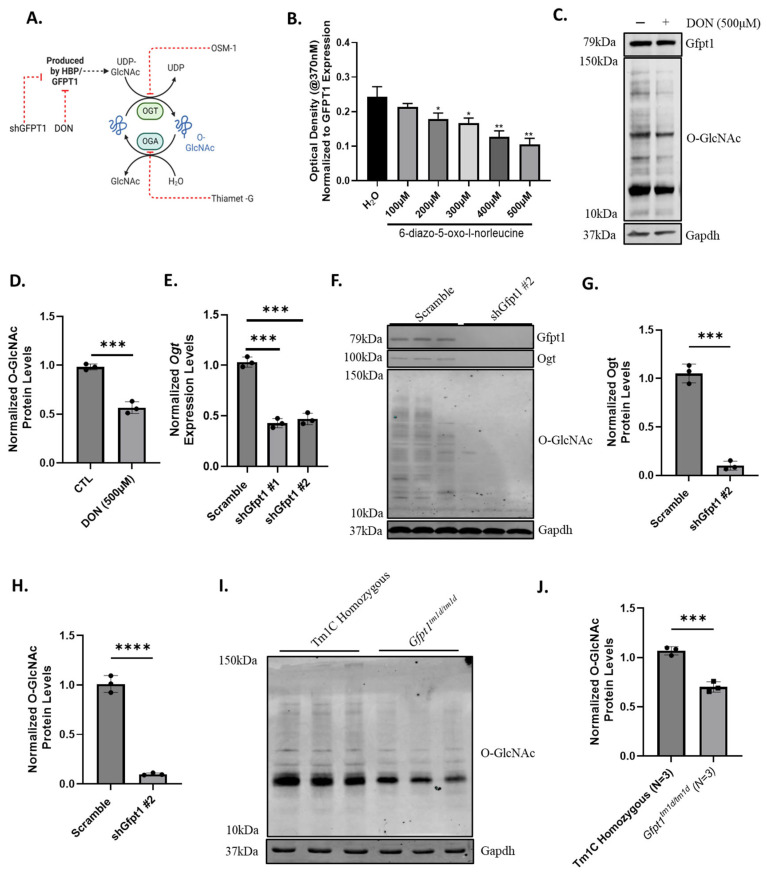
Impairment to GFPT1 activity or protein levels reduces protein O-GlcNAcylation in vitro and in vivo. (**A**) UDP-GlcNAc is a product of the HBP that is required for O-GlcNAcylation. Ogt is used to transfer the GlcNAc moiety to the target protein, which can be reversed by Oga. These enzymes work in tandem to control the level of protein O-GlcNAcylation inside the cell. (**B**) The Gfpt1 antagonist, DON, provided a dose-dependent decrease in Gfpt1 enzymatic activity in wild-type C2C12 cells. (**C**) Western blot of Gfpt1 protein and O-GlcNAc protein modification levels following treatment with DON in wild-type C2C12 cells. (**D**) DON treatment reduced O-GlcNAc modification levels in wild-type C2C12 cells. (**E**) Gfpt1 deficiency reduces Ogt gene expression in C2C12 myotubes. (**F**) Gfpt1, Ogt, and O-GlcNAc modification protein levels assessed via Western blot in Gfpt1-deficient C2C12 cells treated with doxycycline. (**G**) Gfpt1-deficient myotubes had reduced levels of Ogt and (**H**) protein O-GlcNAc modification after treatment with doxycycline. (**I**) A Western blot was used to examine O-GlcNAc levels in *Gfpt1^tm1d/tm1d^* and Tm1C homozygous control mice in quadriceps muscle. (**J**) *Gfpt1^tm1d/tm1d^* mice exhibit a reduction in O-GlcNAc protein modification levels upon comparison with Tm1C homozygous control mice isolated quadriceps. Original images can be found in Supplementary File S1. Graphs are mean ± SD, *n* = 3 for all experiments, and statistical significance was determined by Student’s *t*-Test. A one-way ANOVA was performed for statistical significance. * *p*-value < 0.05, *p*-value ** < 0.01, *p*-value *** < 0.001 and **** *p*-value < 0.0001.

UDP-GlcNAc, the downstream product of the HBP, is essential for the formation of N- and O-linked glycan structures, which are crucial for maintaining protein stability. In addition, UDP-GlcNAc can be used for protein O-GlcNAcylation, a small modification that is essential for cellular signaling (Figure 5A). OGT, an enzyme that transfers GlcNAc to protein moieties, regulates this reversible modification, along with the O-linked glycanase enzyme called OGA, which removes GlcNAc. We hypothesized that impairment to GFPT1/Gfpt1 expression and/or activity would result in reduced O-GlcNAcylation levels. Firstly, we used the Gfpt1 antagonist 6-diazo-5-oxo-l-norleucine (DON) to inhibit enzymatic activity in vitro. Through a dose–response analysis, we confirmed that Gfpt1 is inhibited without causing cellular toxicity in C2C12 cells at 500 μM of DON for 48 h (Figure 5B). Subsequently, we examined the levels of O-GlcNAcylation after DON treatment and showed a significant reduction in protein O-GlcNAcylation (Figure 5C,D). Additionally, treating cells with the Oga inhibitor, Thiamet-G, increased O-GlcNAcylation in wild-type C2C12 cells (Supplementary Figure S7A,B). These results collectively indicate that Gfpt1 enzymatic activity is involved in O-GlcNAcylation of C2C12 cells in vitro.

Next, we wanted to determine if our Gfpt1-depleted cell models have reduced Gfpt1 enzymatic activity, resulting in reduced protein O-GlcNAcylation levels. Gfpt1-depleted C2C12 myoblasts exhibited altered Gfpt1 enzymatic activity (Supplementary Figure S7C). We found that Ogt levels were secondarily reduced in Gfpt1-depleted C2C12 myoblasts, indicating impaired output from O-GlcNAcylation as a result of reduced substrate availability (Figure 5E). To confirm these findings, we examined protein levels of Ogt and O-GlcNAcylation in Gfpt1-depleted C2C12 myoblasts and observed severe depletion in both (Figure 5F,G, Supplementary Figure S7D,E). We also used the lectin succinylated wheat germ agglutinin (sWGA), which has an affinity for N-acetylglucosamine but not sialic acid and has been commonly used to observe alterations in O-GlcNAcylation levels with accompanying O-GlcNAc antibodies. Western blot was used on Gfpt1-deficient lysates, which observed a significant decrease in the amount of sWGA bound to glycoproteins compared to controls, further suggesting that glycosylation impairment is present (Supplementary Figure S7F,G).

Lastly, we examined if there was depleted O-GlcNAcylation in our *Gfpt1^tm1d/tm1d^* mouse model. We observed a reduction in protein O-GlcNAcylation levels in skeletal muscle derived in Gfpt1^tm1d/tm1d^ mice (Figure 5H,I). Additionally, using sWGA, we examined the amount of glycoprotein present within the *Gfpt1^tm1d/tm1d^* skeletal muscle and observed a significant decrease (Supplementary Figure S7H,I). These results collectively indicate that Gfpt1 depletion impairs protein glycosylation, which may be an important mechanism for Gfpt1-CMS by impacting the quality and abundance of NMJ-associated proteins. In addition, these results indicate that the impairment of O-GlcNAcylation likely affects many proteins at the NMJ and in skeletal muscle. However, further functional studies are needed to finally confirm if the AChRδ is O-GlcNAcylated.

## 4. Discussion 

GFPT1 is the rate-limiting enzyme of the HBP, a metabolic pathway that produces UDP-GlcNAc, a key precursor for protein glycosylation [39,40]. Biallelic mutations to *GFPT1* cause CMS, a rare neuromuscular disease characterized by fatigable muscle weakness [21,22,24,25,26,27,28,29]. These mutations to *GFPT1* reduce enzymatic activity and protein expression within the skeletal muscle, decreasing the production of UDP-GlcNAc, an essential precursor for protein glycosylation [22,28,51]. Glycosylation is a PTM that attaches sugar moieties to target proteins at various amino acids, allowing for enhanced solubility and stability. At the NMJ, glycosylation is an essential modification that controls the stability of key receptor complexes both in the pre- and post-synaptic regions [60,63]. It remains unclear how an enzyme essential for protein glycosylation, which is ubiquitously expressed in the body, impairs neurotransmission and NMJ maintenance in GFPT1-related CMS. We hypothesized that a deficiency of Gfpt1 would result in the improper glycosylation of key NMJ-associated proteins impairing function and/or stability within the NMJ and thus analyzed murine in vivo and in vitro models depleted or even deficient for Gfpt1 in skeletal muscle cells. 

We demonstrate that there is a glycosylation deficiency within Gftpt1-depleted models and that these models express an elevated amount of an immature glycoprotein of the AChRδ subunit. We show that both in vitro and in vivo models show a significant reduction in total AChRδ protein levels. We also identified a low-molecular-weight species that was more prominent within *Gfpt1^tm1d/tm1d^* skeletal muscle. PNGase F treatment caused a molecular weight shift in both protein species, indicating that the low-molecular-weight species is an immature glycoprotein. We wanted to determine if this immature glycoprotein is localized to the NMJs of *Gfpt1^tm1d/t1d^* mice as part of the assembled AChR. LCM was used to excise NMJs from skeletal muscle in the Tm1C homozygous control and *Gfpt1^tm1d/tm1d^* mice. We identified that both species were present within the NMJs of *Gfpt1^tm1d/tm1d^* mice, suggesting that deficiently glycosylated AChRδ is localized within proximity to the NMJ. Furthermore, we showed that in both in vitro and in vivo models, reduced Ogt protein levels were present, which resulted in a decrease in total O-GlcNAcylation. Also, succinylated-WGA, a lectin with an affinity for O-GlcNAc residues [64], showed reduced expression in *Gfpt1^tm1d/tm1d^* skeletal muscle. Lastly, we also showed that terminal glycosylation through poly-sialylation was reduced in *Gfpt1^tm1d/tm1d^* skeletal muscle, suggesting that, in totality, several glycan chain assembly pathways are impaired. 

The AChRδ is a member of the pentameric muscle nicotinic AChR, a ligand-gated ion channel with affinity for ACh. The AChR is regulated by glycosylation at the γ and δ subunits, allowing for enhanced stability and trafficking toward the post-synaptic membrane [61]. The AChRδ contains three glycosylation sites: Asn76, Asn143, and Asn169 [60]. Site-directed mutagenesis has previously caused similar molecular weight shifts when expressed in COS cells, suggesting that our low-molecular-weight species is an immature glycoprotein [60]. In addition, these glycosylation-deficient mutants of the AChRδ subunit, when co-expressed with AChRα, β, ε subunits, had impaired AChR receptor assembly and reduced surface expression [60]. It was previously shown that AChRδ and AChRβ contain novel motifs with lysine residues that enable Golgi retention, promote ubiquitination, and contribute to intracellular retention [61]. As such, this immature glycoprotein may be a target for protein degradation through the proteasomal degradation pathway, a pathway previously shown to regulate the expression of AChRδ through its interaction with the RING domain of Rapsyn [65,66]. Our data suggest that in Gfpt1-deficient samples, an immature glycoprotein of AChRδ is localized to the NMJ. In addition, impairment of glycosylation may impair AChR stability through a similar mechanism, as outlined above. 

Young *Gfpt1^tm1d/tm1d^* mice were previously shown to have phenotypic changes as well as NMJ abnormalities consistent with Gfpt1-related CMS [25]. In this study, we used 40-week-old mice to better identify the molecular changes, anticipating these would be amplified in older animals. We found that *Gfpt1^tm1d/tm1d^* mice have a progressive impairment of muscle endurance and increased fatigability of grip strength. An aged *Gfpt1^tm1d/tm1d^* mouse was also found to have reduced CMAP amplitudes, compared to the control, likely a result of impairment to the skeletal muscle, nerve, and/or NMJ within the hindlimbs [67]. Importantly, *Gfpt1^tm1d/tm1d^* exhibited a fatigable decrement exceeding 15% after low-frequency (3Hz) and high-frequency (20Hz and 30Hz) RNS. In patients with CMS, a decrement greater than 10% is typically observed [59,68,69]. Given this decrement seen with RNS in *Gfpt1^tm1d/tm1d^* mice, this technique could be used as a prognostic marker for therapeutic strategies in the future. However, more samples would be required for full analysis. Similar to our previous paper, *Gfpt1^tm1d/tm1d^* NMJs were small and fragmented, with less complexity compared to Tm1C homozygous control mice [25]. This has also been reported in other models with deficient Gfpt1 expression. One study targeted the muscle-specific 18 amino acid insertion within human GFPT1, termed Gfpt1-L [23,70]. Loss of Gfpt1-L revealed impaired motor performance and NMJ integrity, which progressively worsened over time [23,70]. In addition, both these models contain tubular aggregates within the skeletal muscle fiber, a hallmark of GFPT1-related CMS [21,24,26,28]. As a result, the *Gfpt1^tm1d/tm1d^* models had progressive skeletal muscle weakness seen in GFPT1-related CMS patients and is consistent with other models. 

Previous studies suggested that global N-linked glycosylation is not altered in Gfpt1-CMS myoblasts [52]. However, our data indicate that Gfpt1 deficiency impairs protein glycosylation of specific proteins through both N- and O-linked pathways. A potential difference between these studies is that *Gfpt1^tm1d/tm1d^* mice exhibit a complete Gfpt1 knockout within skeletal muscle, while patients observe a deficiency in expression [22,24,28,29,51]. This could exacerbate the issues with glycosylation within our tissue compared to patient controls. To this end, Gfpt1-depleted C2C12 cells were prepared, which expressed a similar knockdown in expression compared to GFPT1-related-CMS patient-derived myoblasts. We identified that the AChRδ subunit was hypo-glycosylated within both our models. The previous study examined global N-linked glycosylation levels, not glycosylation of an individual protein. Therefore, the occupancy of glycosylated glycan side chains was not examined in that previous study. Our study builds upon this research by highlighting, for the first time, that an individual protein, the AChRδ subunit, was hypo-glycosylated within both Gfpt1-deficient models, thus introducing the first target protein of perturbed glycosylation for this subtype of CMS. In addition, the impact of total N-linked glycosylation may not be a global reduction in one specific glycan chain; perhaps various glycoproteins are individually improperly glycosylated, leading to neurotransmission impairment. This explanation would be appropriate given that GFPT1-related CMS patients have limited total body clinical manifestations, as seen with CDG patients [4,26]. Importantly, these results indicate that future studies should examine Gfpt1-related CMS and other glycosylation-based disorders within the context of glyco-proteomics to examine an individual protein change as opposed to global glycosylation impairment. This could reveal other target glycopeptides that could be hypo-glycosylated similarly, as we found with the AChRδ subunit in Gfpt1-depleted or even deficient models.

We identified that in Gfpt1-deficient models, terminal sialyation was also reduced. Sialyation is a terminal modification of proteins that can be highly unstable under mass spectrometric analyses. There have been several advancements in recent years to enhance the detection of glycan chains within different specimens, one of which has been the detection of terminal sialyation, an often unstable glycan modification that is produced from UDP-GlcNAc [50,71,72]. To date, the purpose of protein sialyation within skeletal muscle and the NMJ remains unknown. Dysfunction within protein sialyation has been associated with the neuromuscular condition called GNE myopathy, caused by a mutation to *GNE* [19,50,71]. The role of protein sialylation within the NMJ is not fully resolved. However, sialic acids play an important role in nerve and skeletal muscle tissue [73]. Within skeletal muscle specifically, proteins such as α-dystroglycan and neprilysin are complex glycoproteins within the sarcolemma. These proteins function by preserving sarcolemma integrity through the coupling of the actin cytoskeleton to extracellular matrix proteins laminin, neurexin, and agrin [74]. Therefore, sialyation impairment could disrupt muscle morphology, which was present in *Gftp1^tm1d/tm1d^* mice. At the NMJ, sialylation appears to have a role in laminin-induced AChR, suggesting another pathway that could impair NMJ integrity [75]. 

Glycosylation is a key PTM within the NMJ and skeletal muscle, so investigation of other NMJ-associated proteins should be considered. Firstly, beyond the AChRδ subunit, the other AChR subunits (α, β, γ, and ε) have been reported to be glycosylated. Inhibiting these glycosylation sites completely by site-directed mutagenesis and/or tunicamycin treatment impaired AChR surface expression and receptor assembly, suggesting that other AChR subunits may be impaired within Gfpt1-deficient samples [76]. Although we found no molecular weight shifts within the AChRα in our models, the other AChR subunits (β, γ, and ε) were not investigated and could be hypo-glycosylated. In addition to the other AChR subunits, other NMJ-associated peptides, such as components of the AChR clustering pathway MuSK, LRP4, RAPSYN [77,78], and Agrin [79], are glycosylated. Recent studies suggest that mesoderm development candidate 2 (Mesdc2) binds to Lrp4, facilitating the glycosylation and cell surface expression of Lrp4, reducing the number of AChR receptor complexes, and decreasing MuSK phosphorylation levels [80]. Also, MuSK glycosylation at amino acid sites N222, N338, and N459 has been attributed to a deficiency in the phosphorylation of MuSk and impairs downstream signaling [63]. As such, glycosylation may hinder MuSK dimerization in the absence of agrin, suggesting that glycosylation may be a control mechanism for MuSK by providing adequate steric hindrance/electrostatic repulsion of MuSK monomers [63]. These proteins should be investigated in future studies. Our data describe that mis-glycosylation of AChRδ is present within Gfpt1-deficient samples. It remains unclear if solely hypo-glycosylation of AChRδ would result in the structural changes at the NMJ. We hypothesize that with advanced glycoproteomic studies and/or further individual protein glycosylation assessment, other key NMJ-associated proteins that factor into function and/or maintenance of the NMJ may be mis-glycosylated, providing further consequences for NMJ maintenance and neurotransmission in terms of a more complex molecular interplay. In addition, this also does not discount that beyond NMJ maintenance and neurotransmission, other proteins and pathways may be dysregulated in *GFPT1*-CMS pathology. 

We identified that in both Gfpt1-depleted cells and deficient skeletal muscle, there is deficient protein O-GlcNAcylation. O-GlcNAcylation is a small modification that primarily regulates nuclear, cytosolic, and cellular organelles such as the mitochondria, cytoskeletal, and endoplasmic reticulum [42,55,58]. O-GlcNAcylation attaches a GlcNAc moiety catalyzed by OGT to serine/threonine residues, which can further undergo phosphorylation, suggesting that there may be extensive cellular pathways implicated in Gfpt1-deficient skeletal muscle [43]. In addition, the knockout of the hexosamine biosynthetic enzymes OGT and/or OGA impeded NMJ morphology and maintenance in *Drosophila* [81]. O-GlcNAcylation is enriched within the synapses, playing an essential role in regulating synaptic and neuronal proteins essential for vesicular trafficking [82]. Thiamet-G, a compound that inhibits OGA, causes enhanced O-GlcNAcylation and suppresses synaptic transmission in hippocampal neurons [83]. To our knowledge, O-GlcNAcylation has not been studied in mammalian NMJs specifically. However, these structures contain an enrichment in glycoproteins, mitochondria, and sub-synaptic nuclei, suggesting that proteins are essential for NMJ maintenance and neurotransmission may be impacted.

Within skeletal muscle, O-GlcNAcylation has been associated with regulating transcriptional control through the beta-catenin/Wnt signaling pathway, which serves as a vital component for muscle and NMJ development [48]. Within neuromuscular disease, increased O-GlcNAcylation was detected within regenerating muscle, atrophic, and vacuolated fibers from muscular dystrophy, myositis, rhabdomyolysis, sporadic inclusion body myositis (s-IBM), and distal myopathy with rimmed vacuoles (DMRV) [84]. This suggests that the regulation of protein O-GlcNAcylation is important for muscle health [55]. Furthermore, O-GlcNAcylation is critical to maintaining the structure of the sarcomere through preserving protein–protein interactions of structural proteins in the cytoskeleton [55]. Preserving and/or restoring O-GlcNAcylation within the skeletal muscle may maintain muscle integrity. Lastly, O-GlcNAcylation is a PTM that regulates protein phosphorylation and has been shown to regulate the activity of various pathways inside the cell [85]. It appears that this crosstalk is essential to control cellular signaling pathways and transcription; thus, impaired O-GlcNAcylation may have deleterious impacts on these target proteins [85]. Therefore, understanding the importance of the crosstalk between O-GlcNAcylation and phosphorylation may lead to additional pathways that are perturbed in *GFPT1*-CMS pathology.

In Gfpt1-CMS, muscle fibers have tubular aggregates, deemed a “hallmark” for disease identification [21,24,26]. The presence of these tubular aggregates has been speculated to be associated with hypo-glycosylation of ORAI1, an essential protein in regulating calcium ion flux within the sarcoplasmic reticulum. This has been hypothesized as ORAI1 hypo-glycosylation has been linked to the development of tubular aggregate myopathy and in limb–girdle CMS patients harboring mutation to DPAGT1 [85]. Also, disorders such as tubular aggregate myopathies arise from mutations within STIM1 and/or ORAI1 proteins, resulting in defective sarcoplasmic reticulum calcium flux [86]. As such, defective glycosylation of key proteins responsible for calcium homeostasis may play a role in the presence of tubular aggregates within GFPT1-related CMS. Alternatively, a recent finding suggested endoplasmic reticulum-associated stress due to hypo-glycosylation attributed to the formation of tubular aggregates within GFPT1-L knock-in mice [70]. In addition, this finding demonstrated aged Gfpt-L knock-in progressive decrement in aged mice [70]. Accumulated ER stress attributed to a worsening phenotype; over time, tubular aggregates were observed, as well as eventual activation of autophagy degradation and apoptosis [70]. This study demonstrated that impairment to Gfpt1 activity is attributed to an ER stress environment due to hypo-glycosylation [70]. As such, more hypo-glycosylated proteins may be mis-localized and present within these tubular aggregates that are beyond NMJ-associated peptides.

Importantly, beyond the skeletal muscle, the NMJ comprises three specific cell types: the skeletal muscle, the motor neuron, and the Schwan cell. The motor neuron is essential for propagating the action potential and release of neurotransmitters into the synaptic cleft. The Schwann cell supports the motor neuron by creating a myelin sheath around peripheral neural axons. Our models within this study solely examine the skeletal muscle. As mentioned above, the *Gfpt1^tm1d/tm1d^* mouse model is a skeletal muscle-specific knockout of Gfpt1, while C2C12 cells are murine muscle myoblasts. Importantly, N- and O-linked glycosylation is essential for the function of critical proteins that are essential for motor neuron and Schwann cell development [87,88,89]. As such, further examination of the role of glycosylation within these cell types may elucidate more mechanisms within GFPT1-related CMS patients.

## 5. Conclusions

In conclusion, this study identified a deficiently glycosylated protein within Gfpt1-deficient models, the AChRδ subunit, a key component that forms the AChR complex. We identified that the AChRδ contains an increase in an immature glycoprotein that is localized to the NMJs of *Gfpt1^tm1d/tm1d^* mice. In addition, we identified that Gfpt1-depleted cells and Gfpt1-deficient tissue have impaired protein O-GlcNAcylation, an abundant modification within the NMJ and skeletal muscle. This study further adds evidence of deficient glycosylation in GFPT1-related-CMS and maintains that other proteins essential for NMJ integrity may be differentially glycosylated, further impairing NMJ maintenance and neurotransmission. 

## Data Availability

Data are contained within the article and Supplementary Materials.

## Supplementary Materials

The following supporting information can be downloaded at: https://www.mdpi.com/article/10.3390/biom14101252/s1, Figure S1: In vivo characterization of the long-term effects of Gfpt1-deficiency; Figure S2: In vivo characterization RNS decrement in *Gfpt1^tm1d/tm1d^* mice; Figure S3: Characterization of AChRδ protein levels in *Gfpt1^tm1d/tm1d^* mice; Figure S4: Production and validation of the doxycycline-inducible Gfpt1-deficient C2C12 cell model; Figure S5: Laser capture microdissection collection of NMJ enriched proteins in C57Bl/6N mice; Figure S6: Laser capture isolation of NMJs from *Gfpt1^tm1d/tm1d^* and Tm1C homozygous control mice; Figure S7: Modulation of hexosamine biosynthetic pathway with Thiamet-G or knockdown of Gfpt1 alters O-GlcNAcylation levels; Table S1: Primer sequences for Rt-qPCR; File S1: All original Western blot images.
