# Supplementary material for: A Deficiency in Glutamine-Fructose-6-Phosphate Transaminase 1 (Gfpt1) in Skeletal Muscle Results in Reduced Glycosylation of the Delta Subunit of the Nicotinic Acetylcholine Receptor (AChRδ)
Source: Biomolecules. 2024 Oct 3;14(10):1252. doi: 10.3390/biom14101252 (PMC11506803; doi:10.3390/biom14101252)
Supplement: Supplementary file 1 [file biomolecules-14-01252-s001.zip › biomolecules-3180052-supplementary-proof.pdf]

This file contains :

- Figures:
  - S1: *In vivo* characterization of the long-term effects of Gfpt1-deficiency.
  - S2: *In vivo* repetitive nerve stimulation: decrement in *Gfpt1*$^{tm1d/tm1d}$ mice.
  - S3: Characterization of AChRδ protein levels in *Gfpt1*$^{tm1d/tm1d}$ mice.
  - S4: Production and validation of the doxycycline-inducible Gfpt1-deficient C2C12 cell model.
  - S5: Laser capture microdissection collection of NMJ enriched proteins in C57bl/6 mice.
  - S6: Laser capture isolation of NMJs from *Gfpt1*$^{tm1d/tm1d}$ and Tm1C homozygous control mice
  - S7: Modulation of hexosamine biosynthetic pathway with thiamet-G or knockdown of Gfpt1 alters in O-GlcNAcylation levels.
- Table S1: Primer sequences for Rt-qPCR

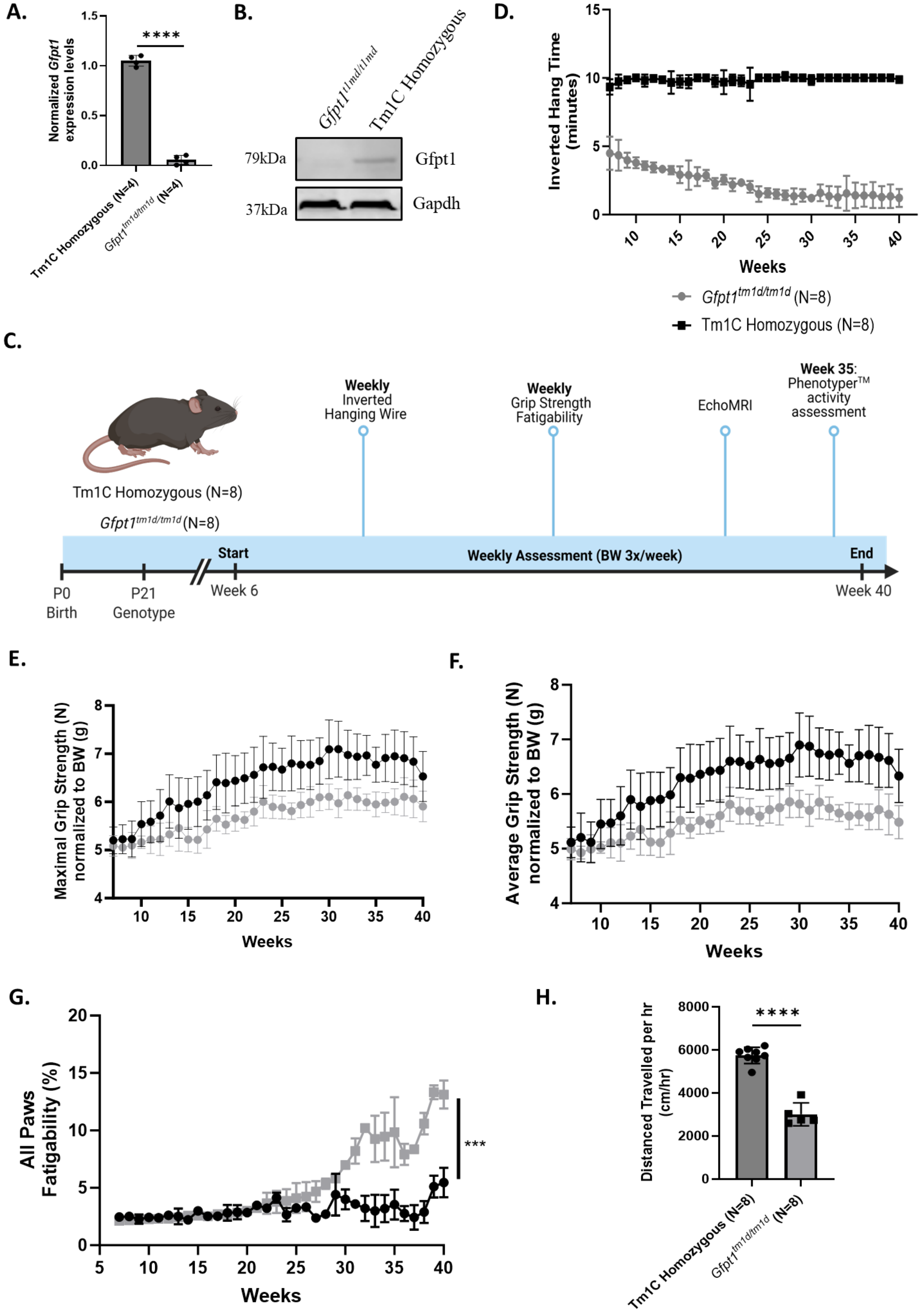
A.
Normalized Gfpt1 expression levels
****
Tm1C Homozygous (N=4)
Gfpt1tm1d/tm1d (N=4)
B.
Gfpt1tm1d/tm1d
Tm1C Homozygous
79kDa
Gfpt1
37kDa
Gapdh
D.
Inverted Hang Time (minutes)
Weeks
Gfpt1tm1d/tm1d (N=8)
Tm1C Homozygous (N=8)
C.
Tm1C Homozygous (N=8)
Gfpt1tm1d/tm1d (N=8)
Weekly Inverted Hanging Wire
Weekly Grip Strength Fatigability
EchoMRI
Week 35: Phenotyper™ activity assessment
Start
Weekly Assessment (BW 3x/week)
End
P0 Birth
P21 Genotype
Week 6
Week 40
E.
Maximal Grip Strength (N) normalized to BW (g)
Weeks
F.
Average Grip Strength (N) normalized to BW (g)
Weeks
G.
All Paws Fatigability (%)
***
Weeks
H.
Distanced Travelled per hr (cm/hr)
****
Tm1C Homozygous (N=8)
Gfpt1tm1d/tm1d (N=8)

Figure S1: ***In vivo*** **characterization of the long-term effects of Gfpt1-deficiency.**

A) RT-qPCR assessment of *Gfpt1* expression showed a significant depletion in *Gfpt1*$^{tm1d/tm1d}$ skeletal muscle compared to Tm1C homozygous control mice. B) *Gftp1*$^{tm1d/tm1d}$ mice exhibit a knockout of Gfpt1 protein (79kDa) within the quadriceps. statistical significance was determined via a two-way ANOVA test where N=8 for both Tm1C homozygous control mice and *Gfpt1*$^{tm1d/tm1d}$ mice.). (Original images can be found in Supplementary File 1.) C) Weekly behavioural assessments were conducted to assess for muscle endurance, strength, and fatigability. These included the inverted hanging wire and repetitive grip strength measurements. Body weight was measured three times per week, EchoMRI measurements were taken every week. A 24hr Phenotyper™ assessment was performed at week 35. statistical significance was determined via a two-way ANOVA test where N=8 for both Tm1C homozygous control mice (black line) and *Gfpt1*$^{tm1d/tm1d}$ mice (grey line). D) The raw inverted hanging wire latency to fall recordings before normalization to mouse body weight. *Gfpt1*$^{tm1d/tm1d}$ mice show a progressive impairment to the latency to fall throughout this study. E) Weekly maximal and F) average grip strength was calculated from *Gfpt1*$^{tm1d/tm1d}$ and Tm1C homozygous control mice. Maximal grip strength was calculated by taking the highest grip strength measurement normalized to body weight. statistical significance was determined via a two-way ANOVA test where N=8 for both Tm1C homozygous control mice and *Gfpt1*$^{tm1d/tm1d}$ mice. G) *Gfpt1*$^{tm1d/tm1d}$ mice exhibit an increase in muscle fatigability compared to Tm1C homozygous control mice using repetitive grip strength measurements from all-paw recordings. statistical significance was determined via a two-way ANOVA test where N=8 for both Tm1C homozygous control mice and *Gfpt1*$^{tm1d/tm1d}$ mice. H) A 24hr Phenotyper™ activity assessment was performed in *Gfpt1*$^{tm1d/tm1d}$ mice and Tm1C homozygous control mice. Measurements were calculated as total distance travelled (cm) per hour (hr). Statistical significance was determined via a Student T-Test. All graphs show mean ± SD. *** $P < 0.001$, **** $P < 0.0001$.

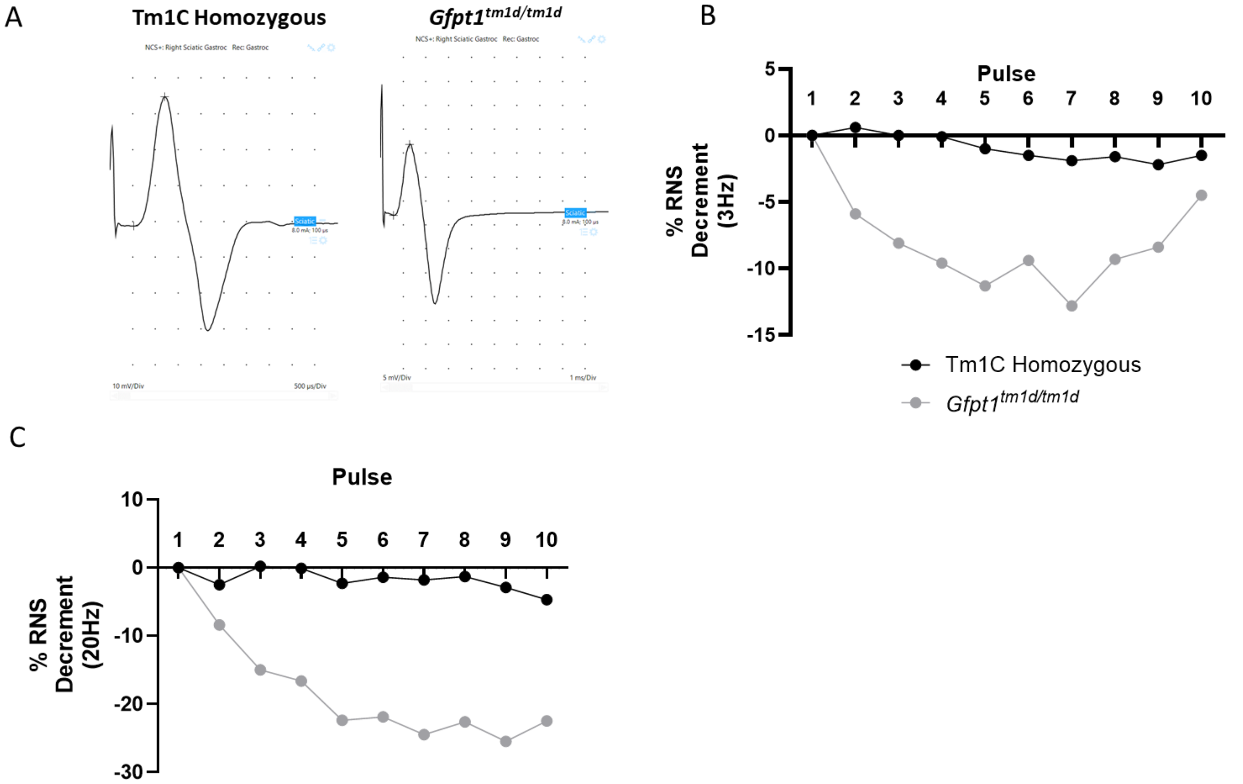

Figure S2: *In vivo* characterization RNS decrement in *Gfpt1*$^{tm1d/tm1d}$ mice.

A) There was a decrease in CMAP amplitude (mV) recorded from the gastrocnemius medialis muscle following repetitive stimulation of the sciatic nerve for both the Tm1C homozygous and Gfpt1$^{tm1d/tm1d}$ mouse. Typically, clinicians look for a decrement between the first and the 7th stimulus and consider a decrement of more than 10% as indicative for neuromuscular transmission defects. In the control mice decrement did not exceed 5% at any of the RNS tests. B) There was a noticeable decrement in Gfpt1$^{tm1d/tm1d}$ skeletal muscle during low-frequency RNS at 3Hz. C) There was a noticeable decrement in Gfpt1$^{tm1d/tm1d}$ skeletal muscle during high-frequency RNS at 20Hz. For all calculations, one *Gfpt1*$^{tm1d/tm1d}$ (Grey line) and one Tm1C homozygous mouse (black line) was recorded at 54 weeks of age before they were sacrificed.

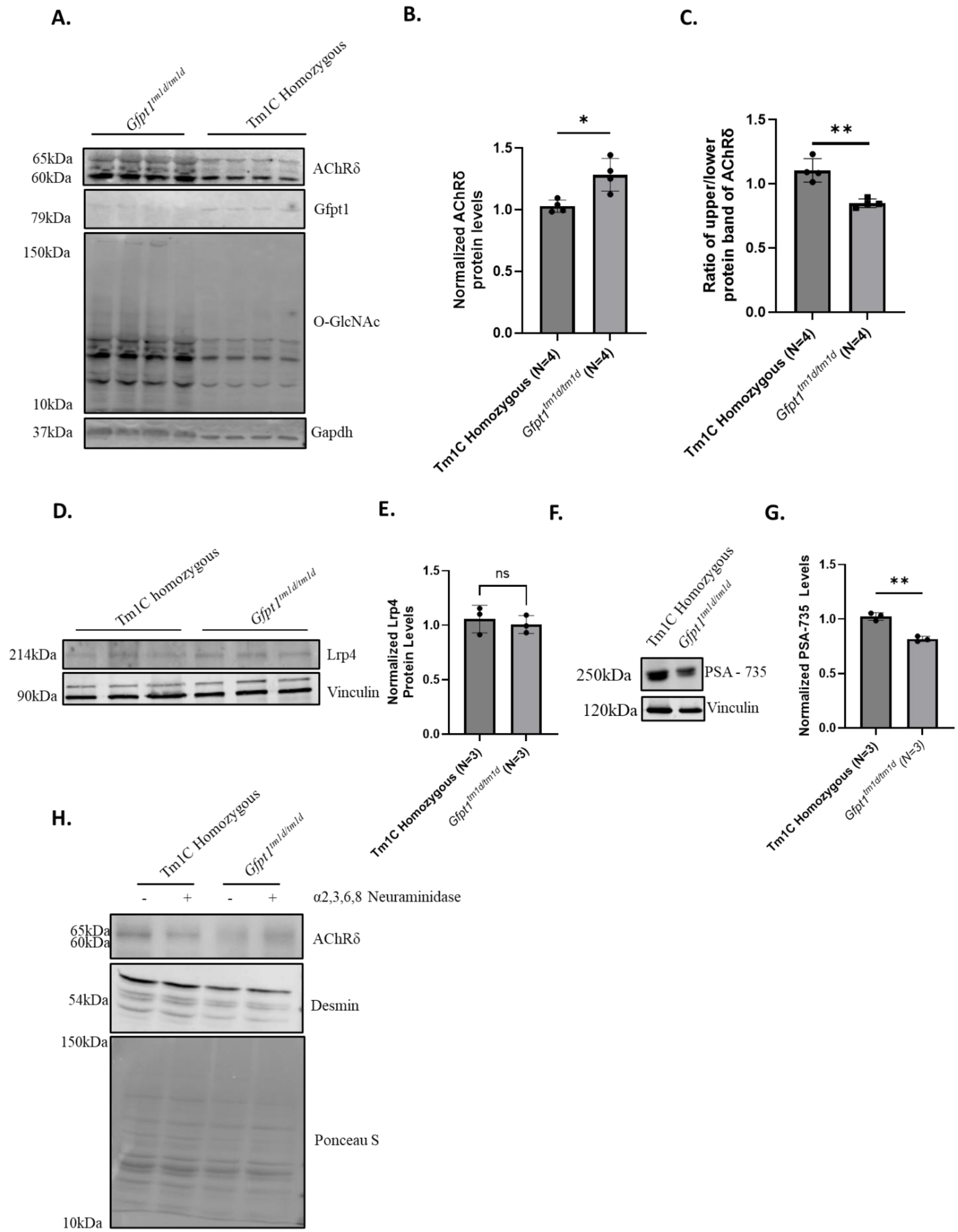
A.
Gfpt1tm1d/tm1d
Tm1C Homozygous
65kDa
60kDa
AChRδ
79kDa
Gfpt1
150kDa
O-GlcNAc
10kDa
37kDa
Gapdh
B.
*
Normalized AChRδ protein levels
1.5
1.0
0.5
0.0
Tm1C Homozygous (N=4)
Gfpt1tm1d/tm1d (N=4)
C.
**
Ratio of upper/lower protein band of AChRδ
1.5
1.0
0.5
0.0
Tm1C Homozygous (N=4)
Gfpt1tm1d/tm1d (N=4)
D.
Tm1C homozygous
Gfpt1tm1d/tm1d
214kDa
Lrp4
90kDa
Vinculin
E.
ns
Normalized Lrp4 Protein Levels
1.5
1.0
0.5
0.0
Tm1C Homozygous (N=3)
Gfpt1tm1d/tm1d (N=3)
F.
Tm1C Homozygous
Gfpt1tm1d/tm1d
250kDa
PSA - 735
120kDa
Vinculin
G.
**
Normalized PSA-735 Levels
1.5
1.0
0.5
0.0
Tm1C Homozygous (N=3)
Gfpt1tm1d/tm1d (N=3)
H.
Tm1C Homozygous
Gfpt1tm1d/tm1d
- + - +
α2,3,6,8 Neuraminidase
65kDa
60kDa
AChRδ
54kDa
Desmin
150kDa
Ponceau S
10kDa

Figure S3: Characterization of AChRδ protein levels in *Gfpt1*$^{tm1d/tm1d}$ mice.

A) AChRδ protein levels (65kDa) were examined in *gastrocnemius* muscle isolated from 40-week-old *Gfpt1*$^{tm1f/tm1d}$ mice and Tm1C homozygous control mice. A 60kDa lower molecular weight species was detected in the *Gfpt1*$^{tm1d/tm1d}$ specimen. B) There was an increase in the 65kDa AChRδ protein levels in the *gastrocnemius* isolated from *Gfpt1*$^{tm1d/tm1d}$ mice. C) The ratio of the upper molecular weight species and the lower molecular weight species was reduced in *Gfpt1*$^{tm1d/tm1d}$ skeletal muscle. D) Lrp4 protein levels (214kDa) were examined in quadriceps muscle isolated from 40-week-old *Gfpt1*$^{tm1f/tm1d}$ mice and Tm1C homozygous control mice. E) There was no significant change in the 214kDa Lrp4 protein levels in the quadriceps isolated from *Gfpt1*$^{tm1d/tm1d}$ mice. F) Total protein poly-sialyation was examined in quadricep muscle isolated from 40-week-old *Gfpt1*$^{tm1d/tm1d}$ and Tm1C homozygous control mice. G) There was a significant reduction in total poly-sialyation level in *Gfpt1*$^{tm1d/tm1d}$ quadriceps. H) To examine sialyation levels of AChRδ quadricep muscle lysates were treated with α2-3,6, 8 Neuraminidase. The molecular weight of AChRδ protein species did not change after treatment. All graphs show mean ± SD, statistical significance was determined via student T-Test. ns $P > 0.05$; * $P < 0.05$, ** $P < 0.01$. (Original images can be found in Supplementary File 1.)

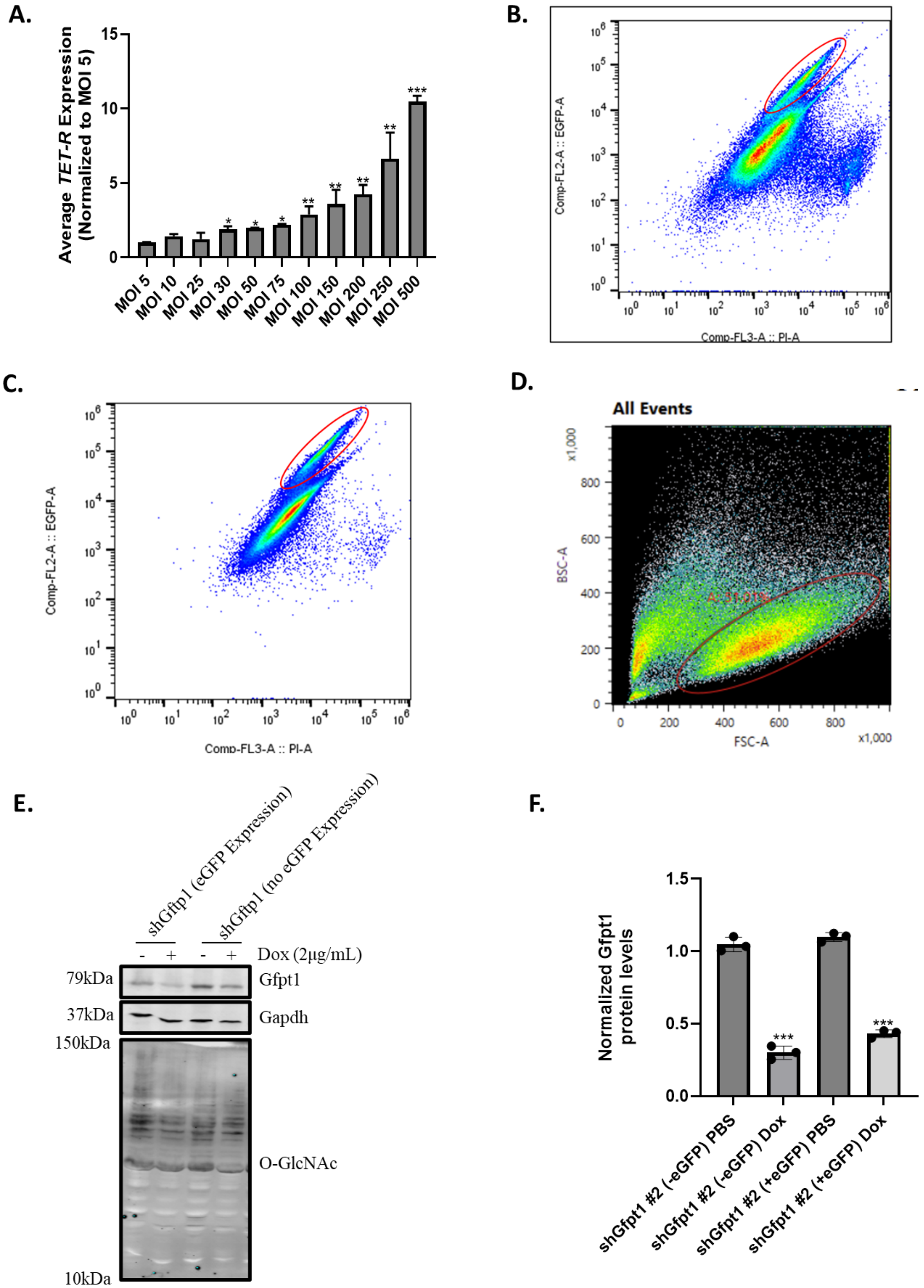
A.
Average TET-R Expression (Normalized to MOI 5)
MOI 5
MOI 10
MOI 25
MOI 30
MOI 50
MOI 75
MOI 100
MOI 150
MOI 200
MOI 250
MOI 500
B.
Comp-FL2-A :: EGFP-A
Comp-FL3-A :: PI-A
C.
Comp-FL2-A :: EGFP-A
Comp-FL3-A :: PI-A
D.
All Events
BSC-A
FSC-A
x1,000
E.
shGfpt1 (eGFP Expression)
shGfpt1 (no eGFP Expression)
Dox (2µg/mL)
79kDa
Gfpt1
37kDa
Gapdh
150kDa
O-GlcNAc
10kDa
F.
Normalized Gfpt1 protein levels
shGfpt1 #2 (-eGFP) PBS
shGfpt1 #2 (-eGFP) Dox
shGfpt1 #2 (+eGFP) PBS
shGfpt1 #2 (+eGFP) Dox

Figure S4: Production and validation of the doxycycline-inducible Gfpt1-deficient C2C12 cell model.

A) TET-R positive colonies of C2C12 myoblasts were assessed for the expression of *TET-R* at different MOIs of lentivirus. This was used to establish whether wild-type C2C12 cells expressed *TET-R* after infection with a TET-R expressing lentivirus and create a polyclonal colony. Statistical test performed was a one-way ANOVA. B) After monoclonal colonies were established post-infection with scramble, shGfpt1 #1 and shGfpt1 #2 lentivirus transgene and treated with doxycycline to activate the expression of eGFP. Then FACS was performed to isolate eGFP positive expressing colonies: scramble, C) shGfpt1 #1, and D) shGfpt1 #2 C2C12 cells. The red circle for all figures encapsulates that cells that were isolated between scramble, shGfpt1 #1 and shGfpt1 #2 populations. E) We noticed that some C2C12 cells had low levels of eGFP expression, these were isolated after cell sorting to establish an eGFP-negative population. To examine Gfpt1 and O-GlcNAc protein modification levels in these cells, treatment with doxycycline was performed in both known populations of eGFP positive and negative C2C12 myoblasts. A Western blot was performed to examine protein levels from Gfpt1 and O-GlcNAc protein levels. (Original images can be found in Supplementary File 1.) F) Notably, regardless of the eGFP expression level, Gfpt1 protein levels were reduced upon activation with doxycycline. Graphs are mean ± SD, statistical significance was determined by Student T-Test. *Denotes a significant difference between scramble and Gfpt1-deficient C2C12 cells, * $P < 0.05$, ** $P < 0.01$. *** $P < 0.001$.

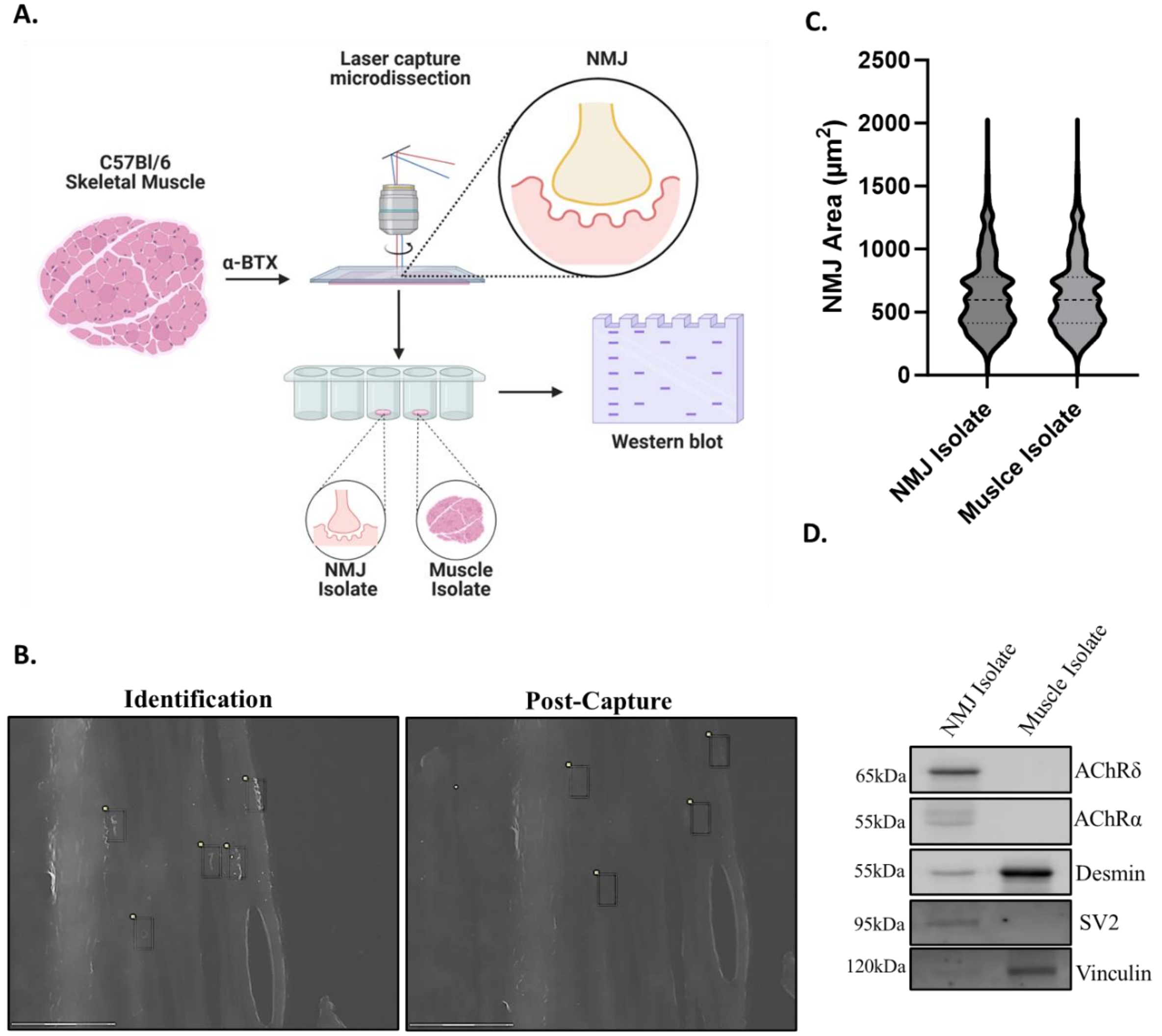

Figure S5: Laser capture microdissection of NMJ enriched proteins in C57bl/6 mice.

A) 25-week-old C57bl/6N mice *gastrocnemius* muscles were longitudinally sectioned and stained for AChR clusters using α-BTX-594. NMJs were identified at 20x magnification and extracted with LCM. Samples were collected for Western blot to assess protein levels. B) A representative image of before and after laser capture microdissection of NMJs from *gastrocnemius* muscles stained with α-BTX-594. Scale bar: 200µm. C) Over 750 NMJs were harvested from 25-week-old C57bl/6N mice. After isolation the exact same amount and size of skeletal muscle (bungarotoxin-negative) was extracted. The above figure describes that the exact same average area of NMJ isolate, and muscle isolate were extracted from the skeletal muscle for analysis. D) The protein levels of NMJ and skeletal muscle markers after LCM enrichment. NMJ post-synaptic markers such as AChRδ, and AChRα, as well as pre-synaptic marker SV2 were enriched in the NMJ isolates and barely detectable in the muscle isolates. Skeletal muscle proteins such as Desmin and Gapdh were detected in both NMJ and skeletal muscle isolates. (Original images can be found in Supplementary File 1.)

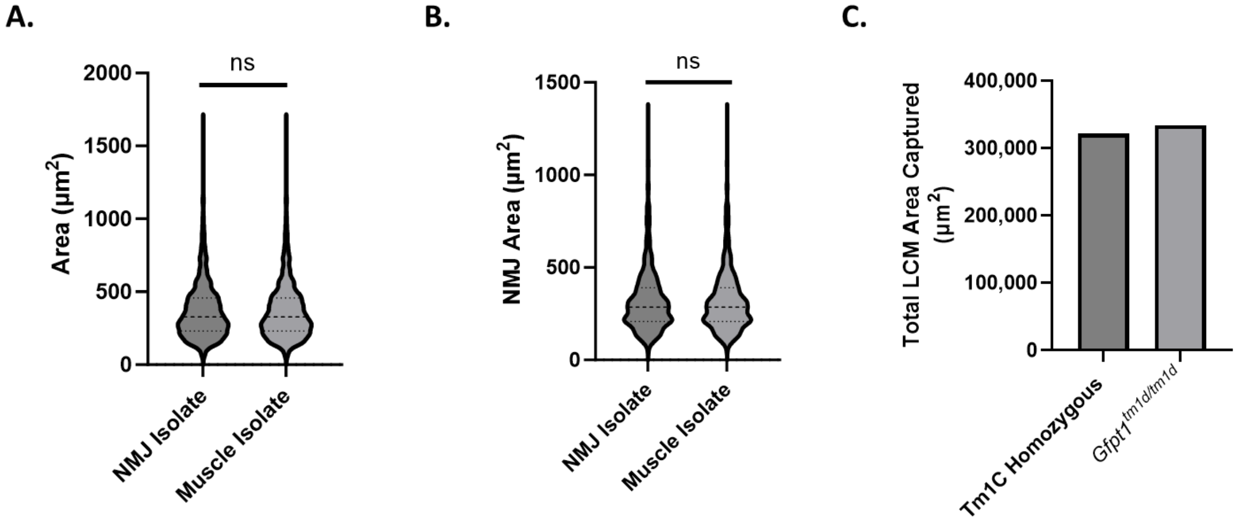

Figure S6: Laser capture isolation of NMJs from *Gfpt1*$^{tm1d/tm1d}$ and Tm1C homozygous control mice

A) For Western blot analysis ~750 NMJs were isolated from Tm1C homozygous control and B) *Gfpt1*$^{tm1d/tm1d}$ muscle stained with α-BTX-594. The average area of the LCM sectioning to represent that there was no change between the NMJ and muscle isolates containing no NMJ material. The data is presented as a violin plot ± SD. C) There was a slight increase in the total LCM area in *Gfpt1*$^{tm1d/tm1d}$ muscle due a smaller AChR area size upon comparison to Tm1C homozygous control muscle. ns Denotes an insignificant difference P value > 0.05.

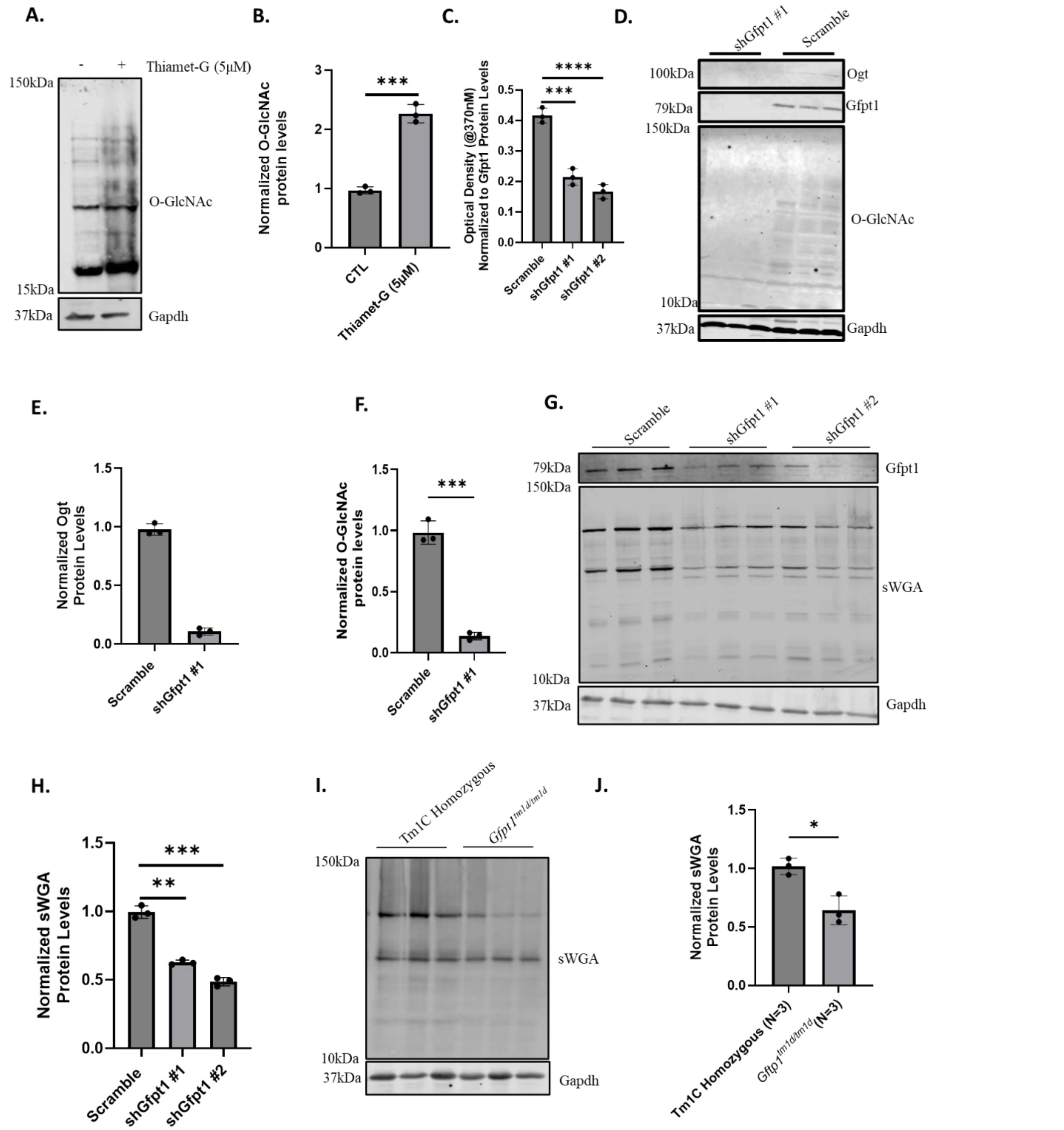

Figure S7: Modulation of hexosamine biosynthetic pathway with thiamet-G or knockdown of Gfpt1 alters in O-GlcNAcylation levels.

A) Western blot assessing wild type C2C12 myotubes treated with 5µM thiamet-G for 72hrs to inhibit the enzymatic activity of Oga, an O-GlcNAcylation glycanase enzyme. B) Thiamet-G treatment increased the O-GlcNAcylation levels in wild-type C2C12 myotubes. C) Enzymatic activity of Gfpt1 was significantly reduced in Gfpt1-depleted C2C12 cells. D) A Western blot examining Gfpt1, Ogt, and O-GlcNAc modification protein levels assessed in scramble, shGfpt1

#1 and infected C2C12 cells treated with doxycycline. E) Ogt and F) O-GlcNAc modification protein levels was reduced in shGfpt1 #1 infected C2C12 myotubes treated with doxycycline upon comparison to scramble infected C2C12 cells. G) A Western blot examining the levels of sWGA lectin expression bound to glycan chains in scramble and Gfpt1-depleted C2C12 cells. H) sWGA lectin binding was significantly reduced in Gfpt1-depleted C2C12 myotubes treated with doxycycline upon comparison to scramble C2C12 myotubes. I) A Western blot examining the levels of sWGA lectin expression bound to glycan chains in the quad isolated from 40-week-old $Gfpt1^{tm1d/tm1d}$ and Tm1C homozygous control mice. J) sWGA lectin binding was significantly reduced the quad isolated from 40-week-old $Gfpt1^{tm1d/tm1d}$ mice. (Original images can be found in Supplementary File 1.) Graphical representations are represented as mean ± SD, statistical significance was determined by student T-Test. * $P < 0.05$ ** $P < 0.01$, *** $P < 0.001$.

Table S1: Primer Sequences for qPCR.

Primer	Primer Sequence
Gfpt1-F	5’- CCA ACG CCT GCA AAA TCC AG-3’
Gfpt1-R	5’-TTC TCC ATG TGT CGC CCA AC-3’
Ogt-F	5’- -3’CTGTCACCCTTGACCCAAA
Ogt-R	5’- -3’ATGGGGTTGCAGTTCGATAG
Chrnd-F	5’- CGC TGC TTC TGC TTC TAG GG -3’
Chrnd-R	5’- ATC AGT TGG CCT TCG GCT T -3’
Chrna-F	5’- CCA CAG ACT CAG GGG AGA TAG
Chrna-R	5’- AAC GGT GGT CTG TGT TGA TGA TG -3’
Gapdh-F	5’- CTC CCA CTC TTC CAC CTT CG -3’
Gapdh-R	5’- GCC TCT CTT GCT CAG TGT CC – 3
Ppia-F	5’- GCC TTC CTC CTT TCA CAG AA -3’
Ppia-R	5’- GAT GCC AGG ACC TGT ATG CT-3’
Rpl27-F	5’- AAG CCG TCA TCG AGA ACA -3’
Rpl27-R	5’- CTT GAT CTT GGA TCG CTT GGC -3’
rtTA-F	5’- CTG GAG TTG AGC AGC CTA C -3’
rtTA-R	5’- AGA GCA CAG CGG AAT GAC TT -3’
