# Supplementary material for: A Deficiency in Glutamine-Fructose-6-Phosphate Transaminase 1 (Gfpt1) in Skeletal Muscle Results in Reduced Glycosylation of the Delta Subunit of the Nicotinic Acetylcholine Receptor (AChRδ)
Source: Biomolecules. 2024 Oct 3;14(10):1252. doi: 10.3390/biom14101252 (PMC11506803; doi:10.3390/biom14101252)

Figure 2C

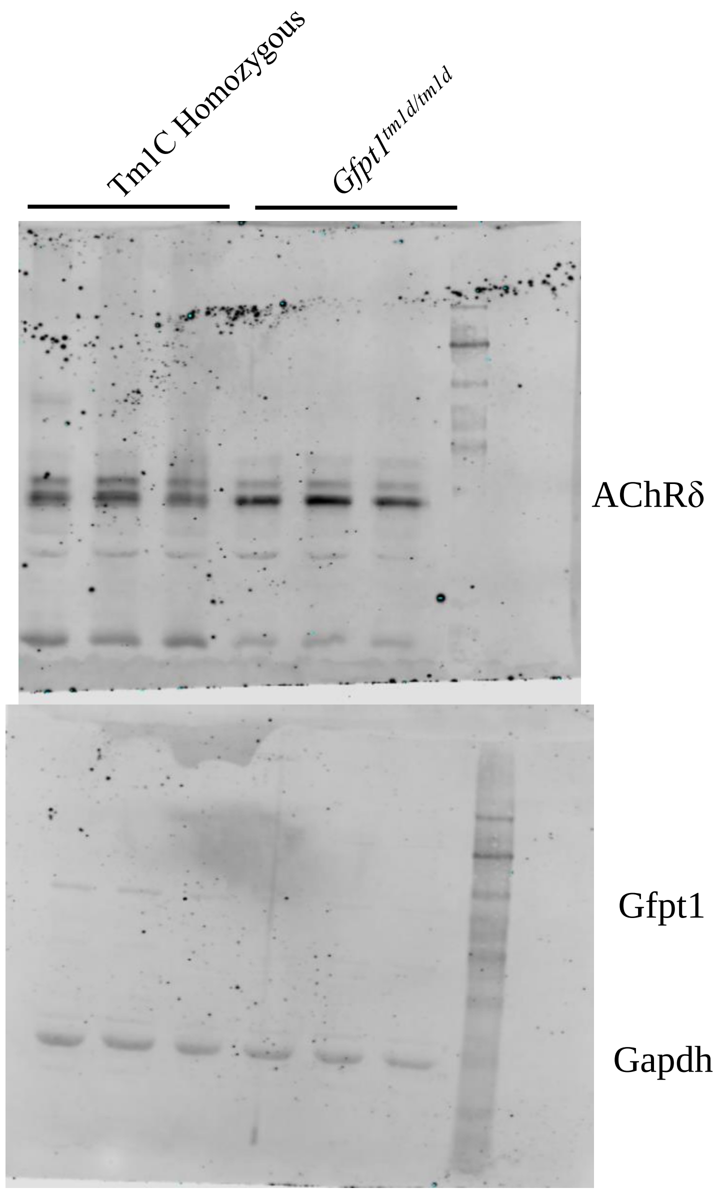

Figure 2F

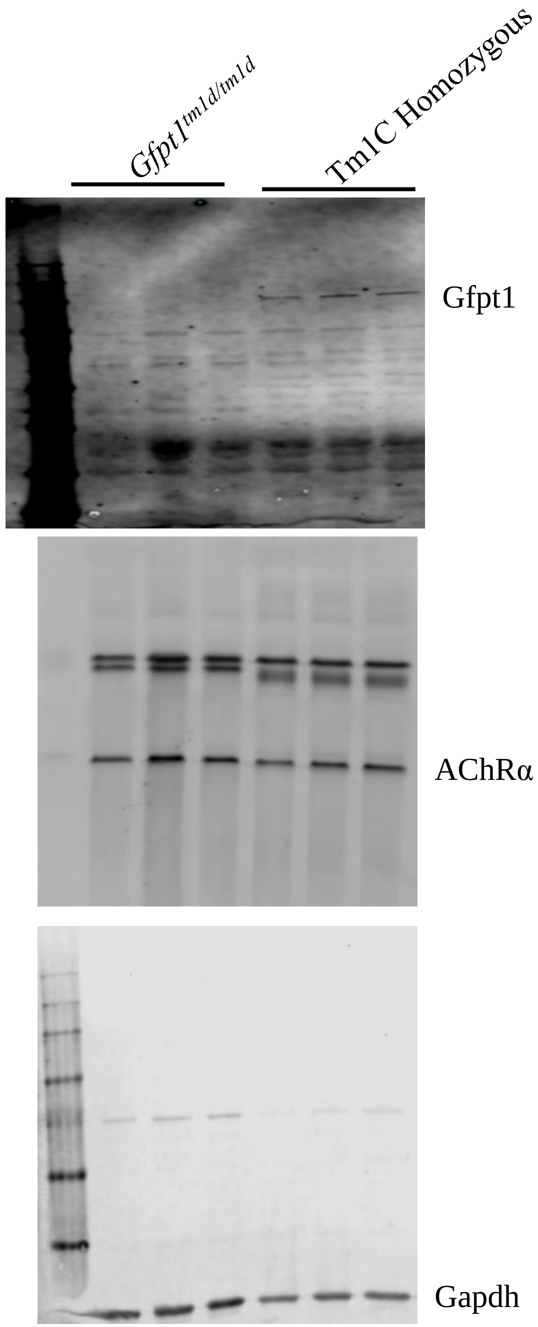

Figure 2H

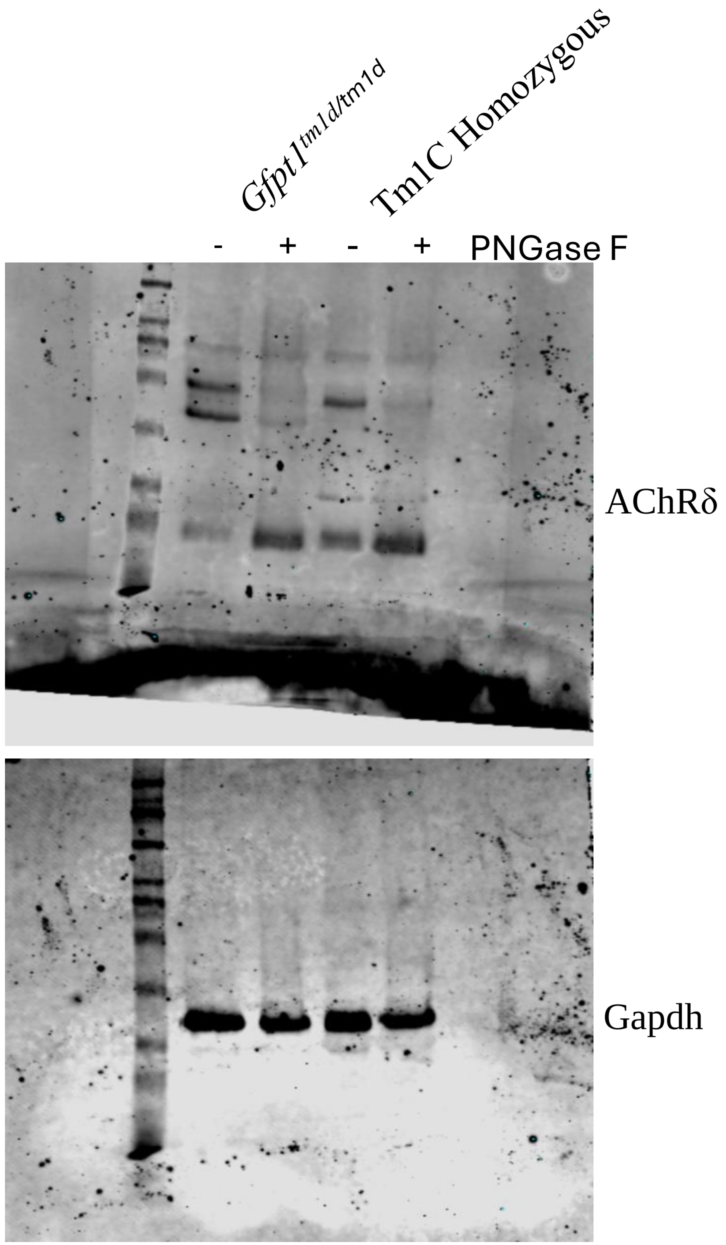

Figure 2I

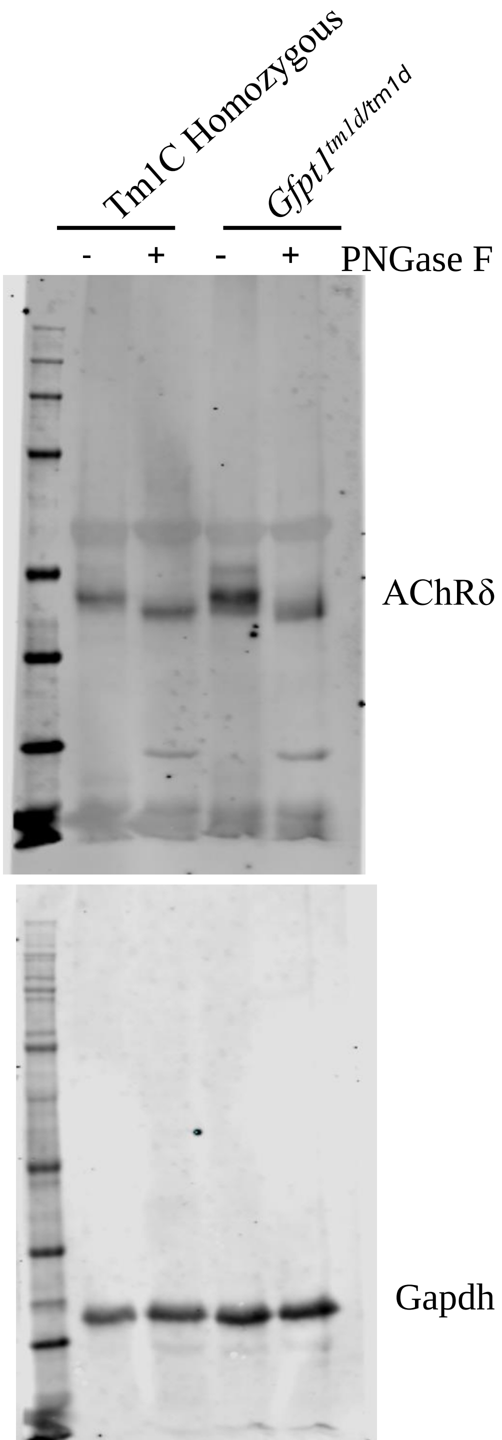

Figure 3C

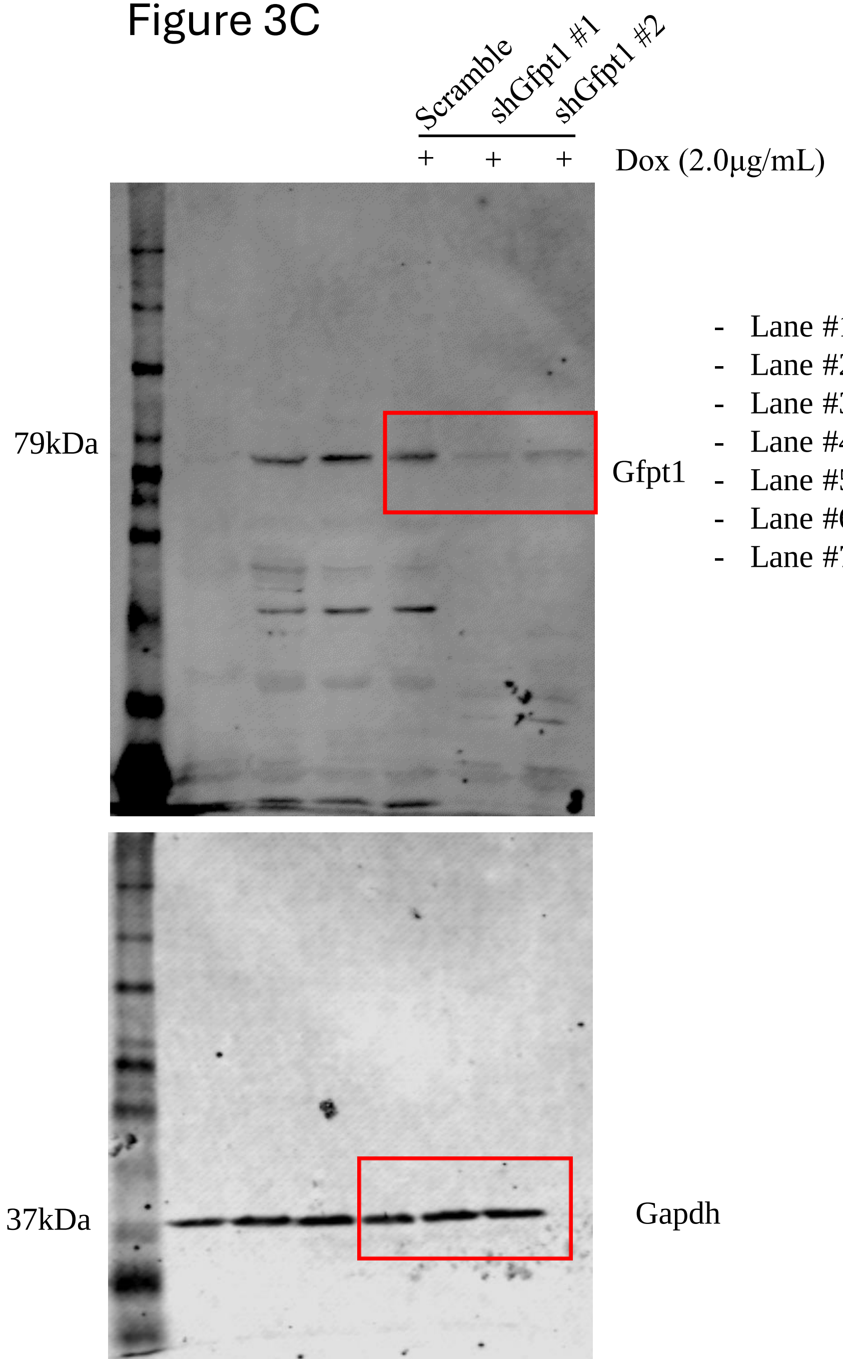

- Lane #1: Ladder
- Lane #2: *Gfpt1$^{tm1d/tm1d}$ quad*
- Lane #3: C57/Bl6 quad
- Lane #4: Tm1C homozygote quad
- Lane #5: Scramble + Dox
- Lane #6: shGfpt1 #1 + Dox
- Lane #7: shGfpt1 #2 + Dox

Figure 3G

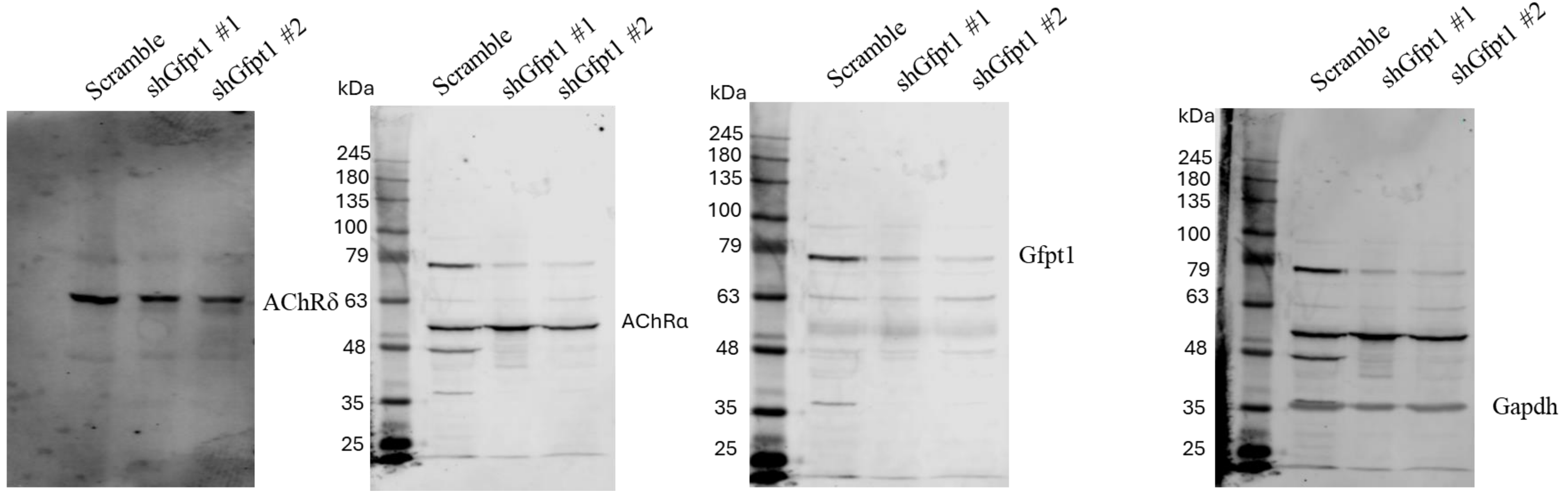
Scramble
shGfpt1 #1
shGfpt1 #2
AChRδ
kDa
245
180
135
100
79
63
48
35
25
Scramble
shGfpt1 #1
shGfpt1 #2
AChRα
kDa
245
180
135
100
79
63
48
35
25
Scramble
shGfpt1 #1
shGfpt1 #2
Gfpt1
kDa
245
180
135
100
79
63
48
35
25
Scramble
shGfpt1 #1
shGfpt1 #2
Gapdh

Figure 4C

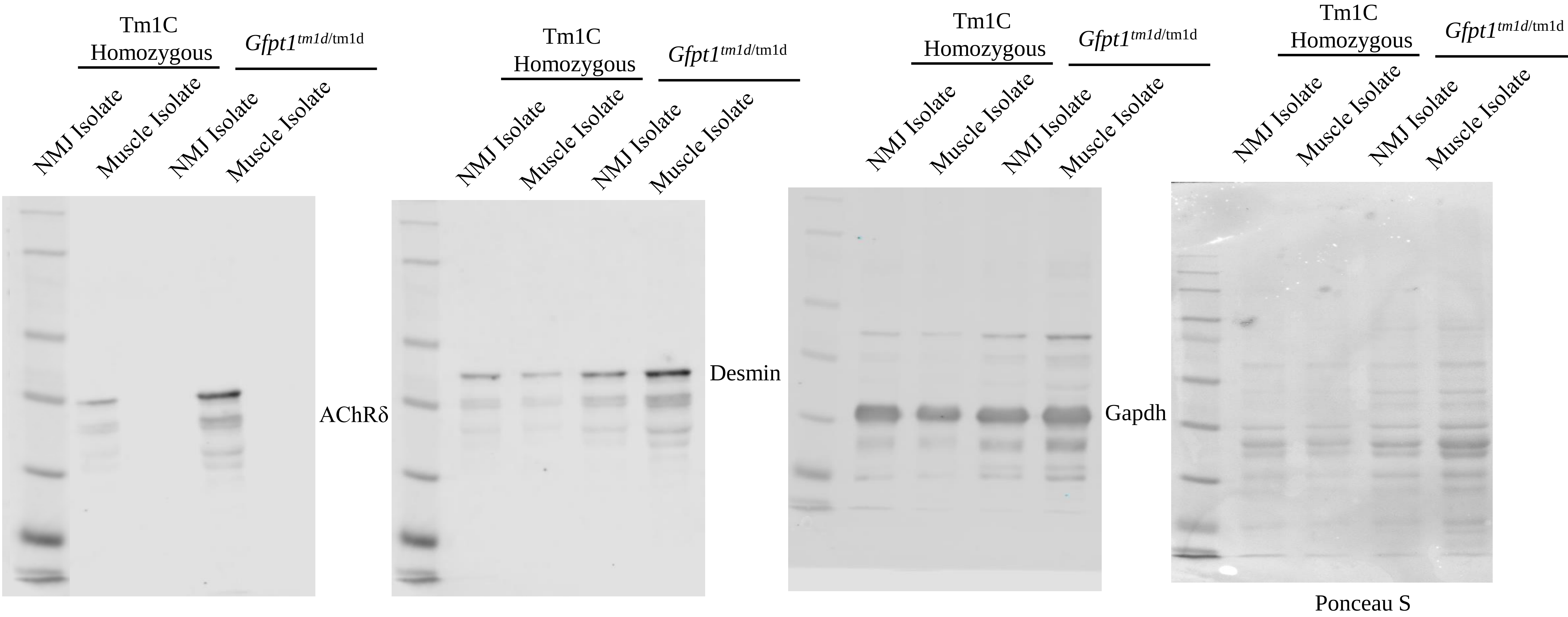

Tm1C Homozygous
$Gfpt1^{tm1d/tm1d}$
NMJ Isolate
Muscle Isolate
NMJ Isolate
Muscle Isolate
AChRδ
Tm1C Homozygous
$Gfpt1^{tm1d/tm1d}$
NMJ Isolate
Muscle Isolate
NMJ Isolate
Muscle Isolate
Desmin
Tm1C Homozygous
$Gfpt1^{tm1d/tm1d}$
NMJ Isolate
Muscle Isolate
NMJ Isolate
Muscle Isolate
Gapdh
Tm1C Homozygous
$Gfpt1^{tm1d/tm1d}$
NMJ Isolate
Muscle Isolate
NMJ Isolate
Muscle Isolate
Ponceau S

Figure 5C

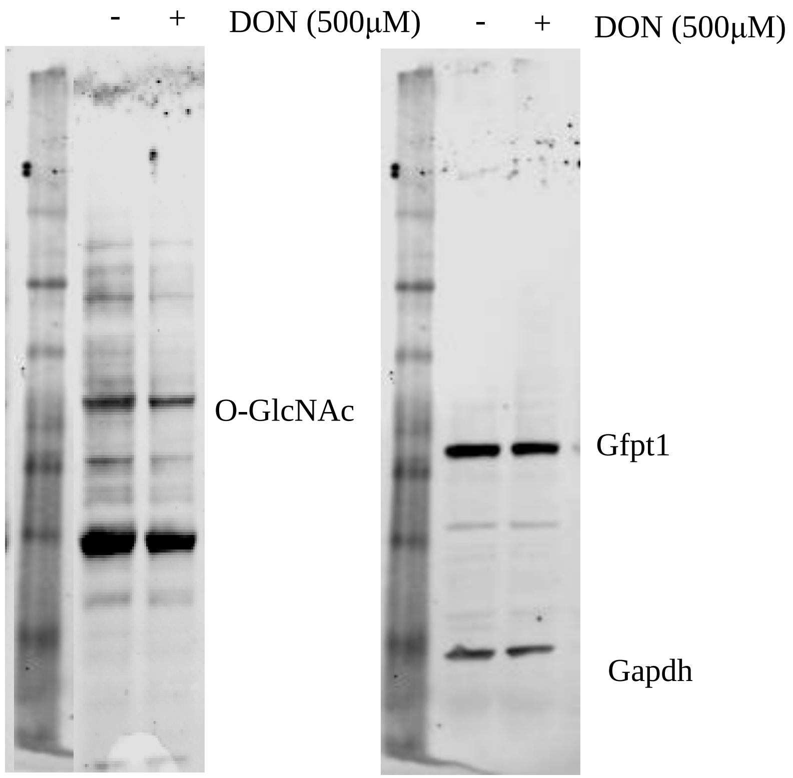

Figure 5F

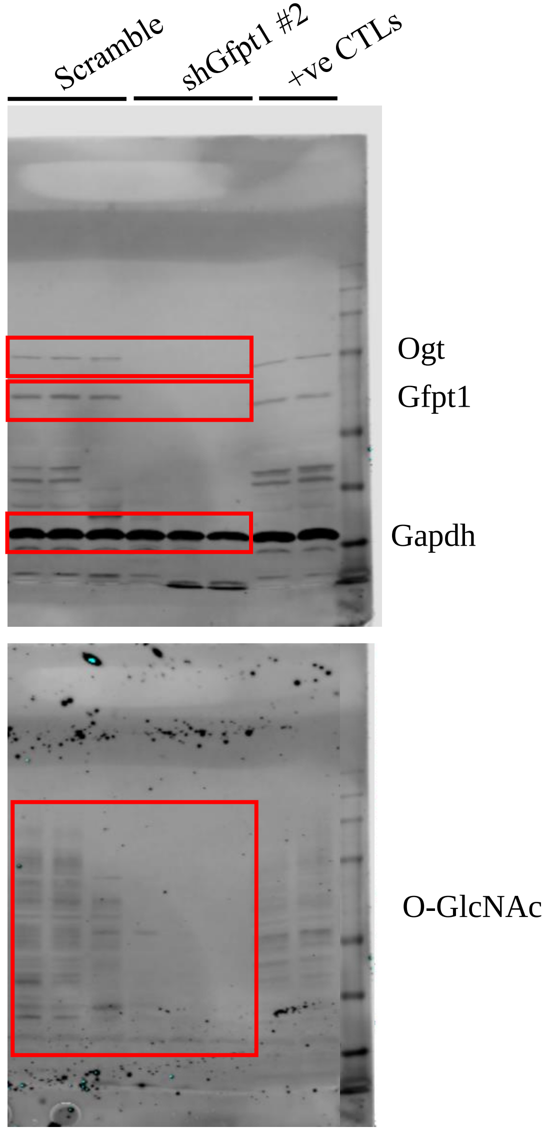

Figure 5I

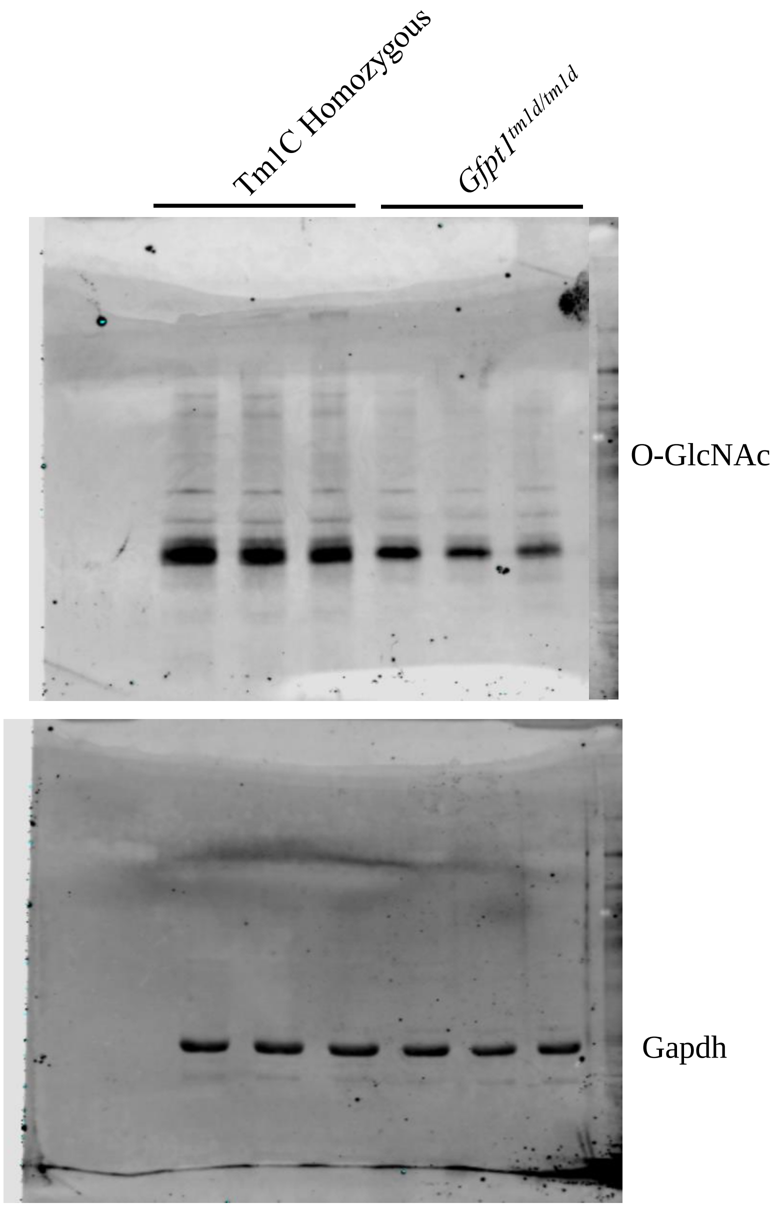

Supplementary 1B

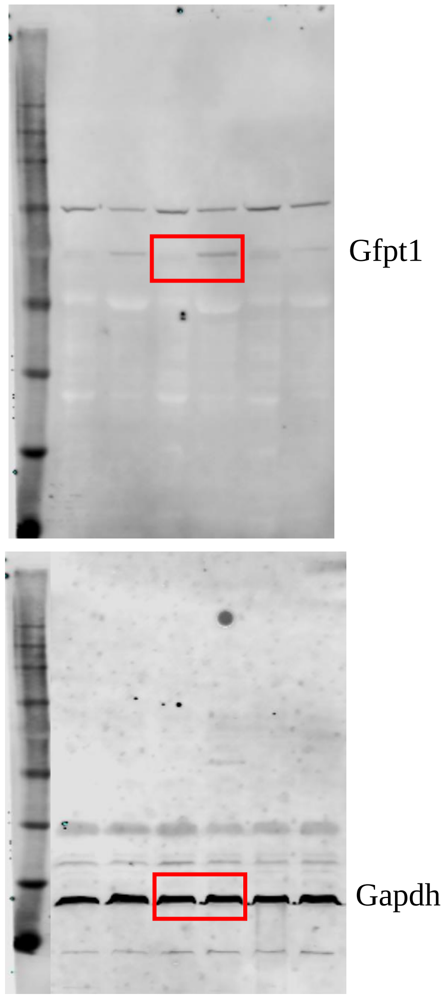

Supplementary 3A

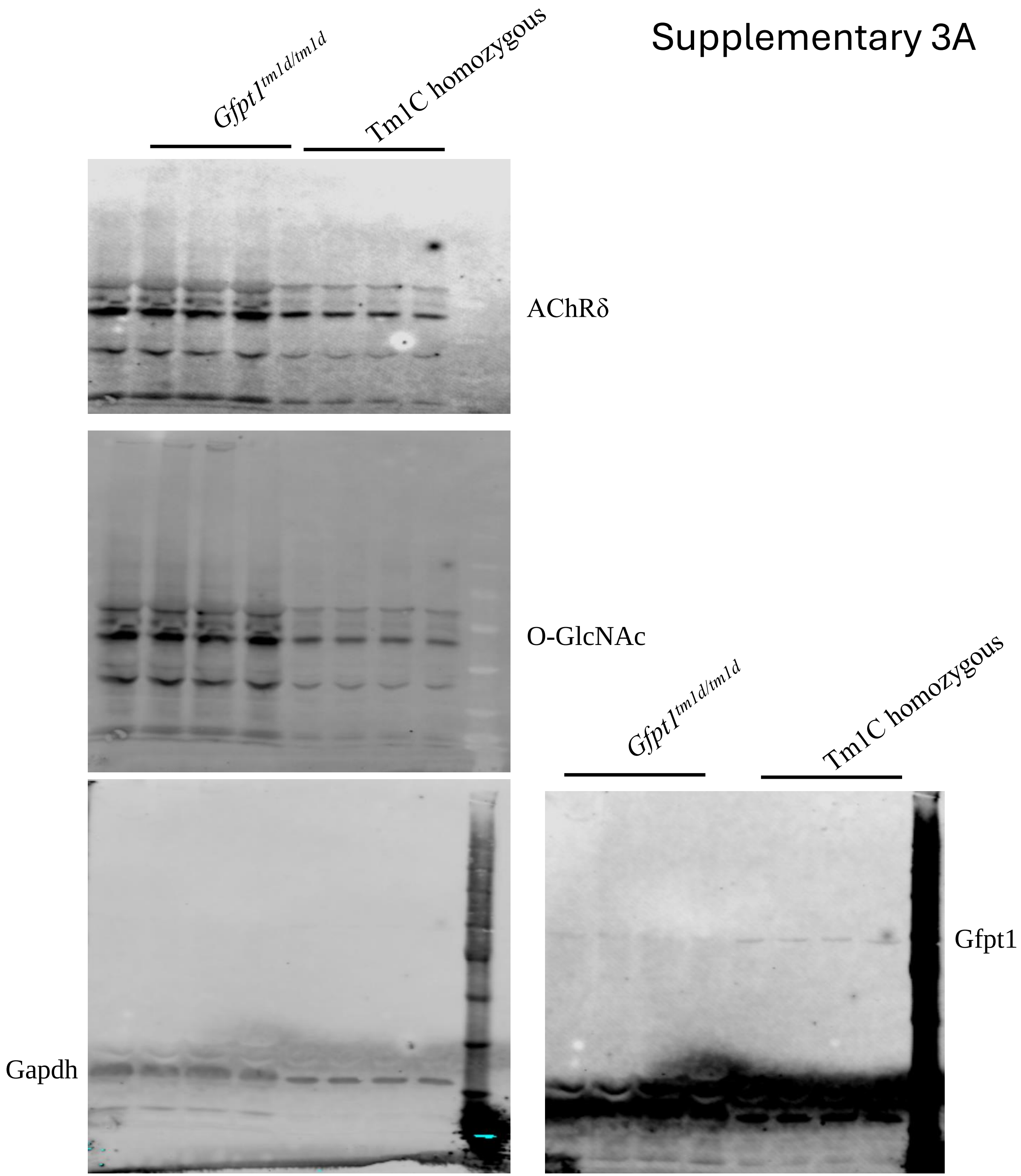

Supplementary 3D

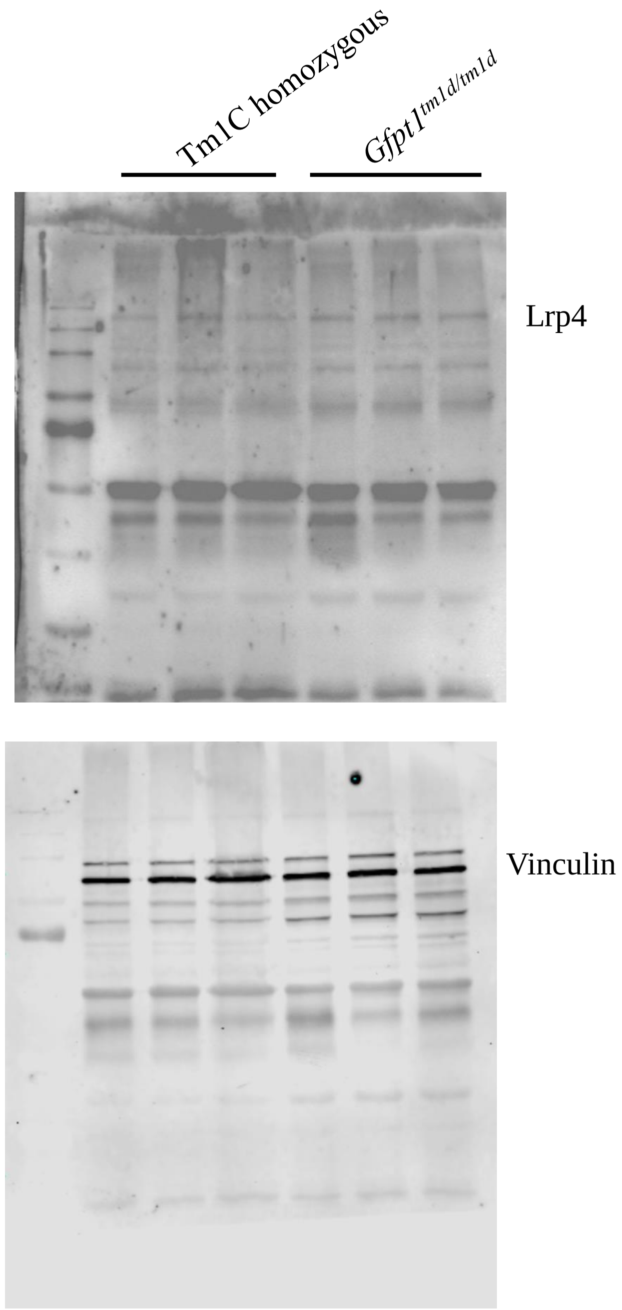

Supplementary 3F

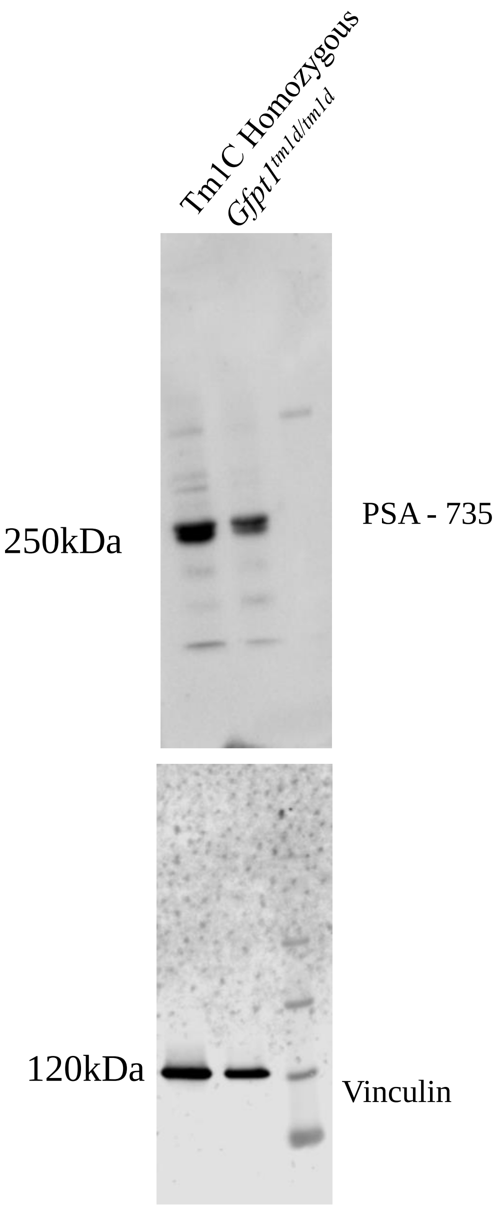

Supplementary 3H

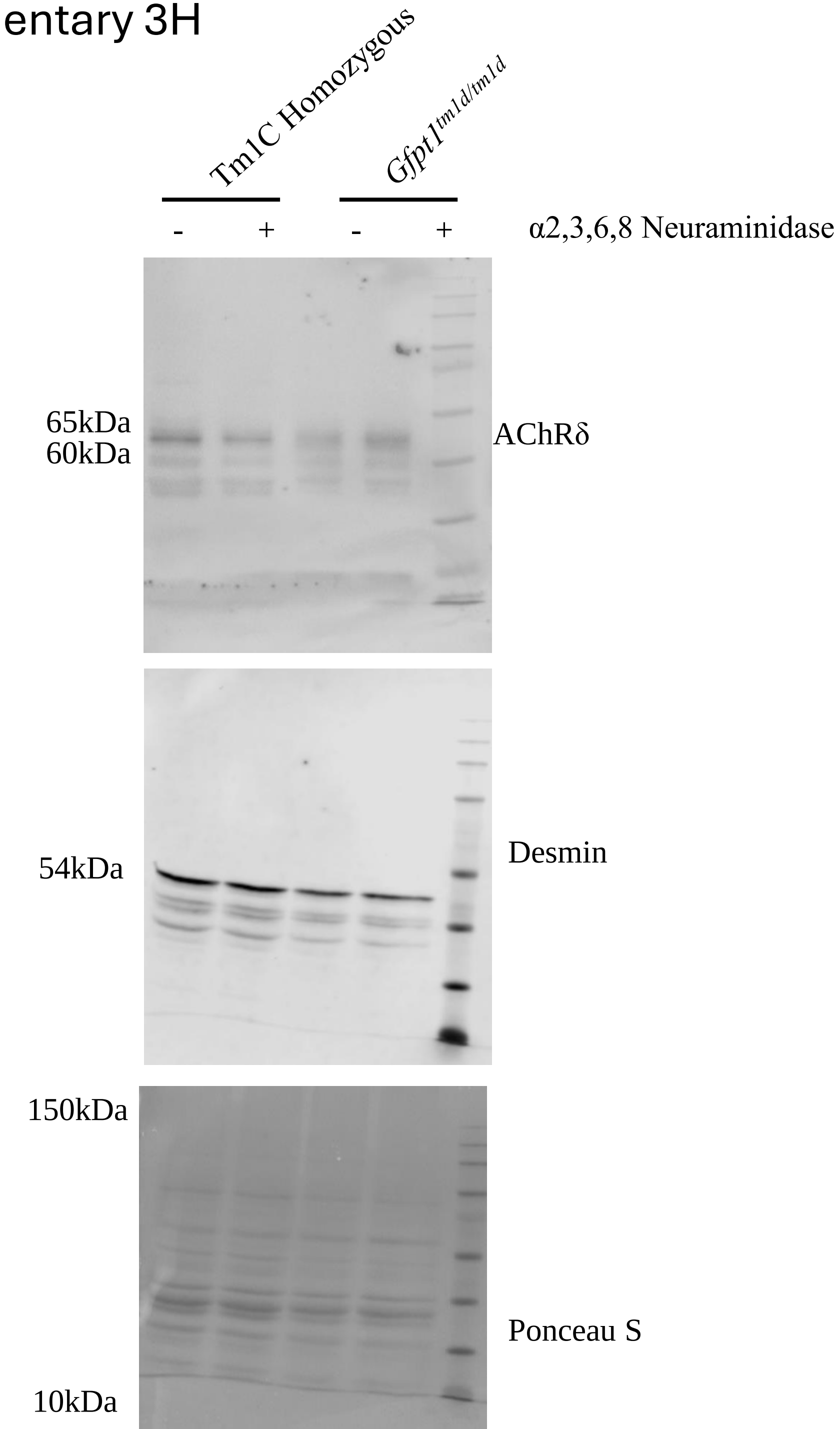

Supplementary 4E

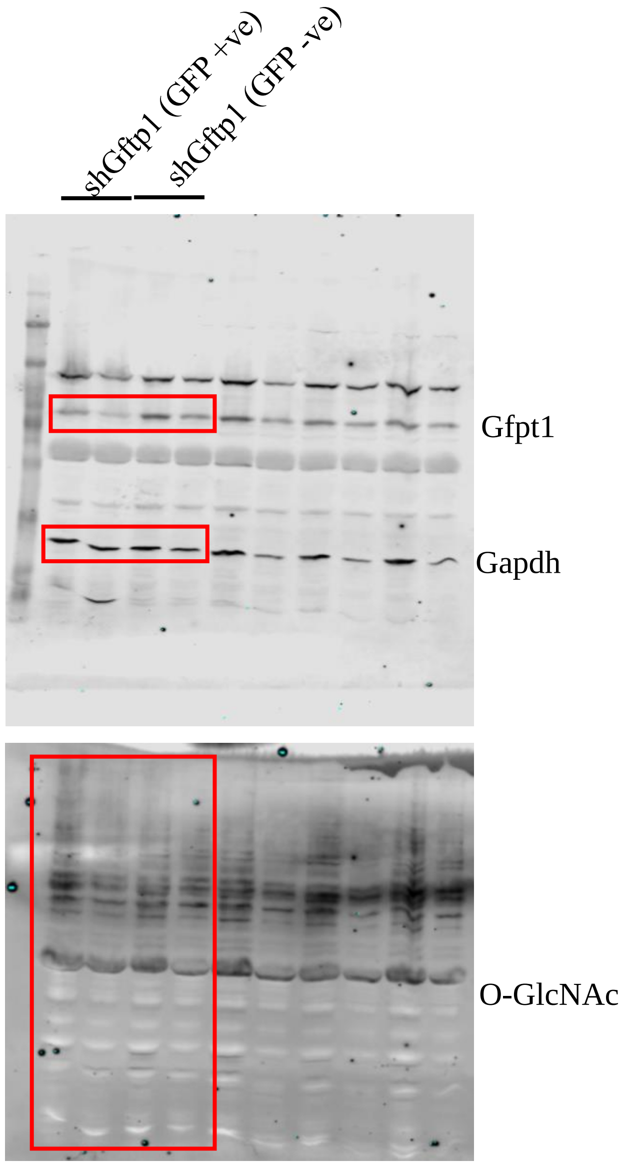

Supplementary 5D

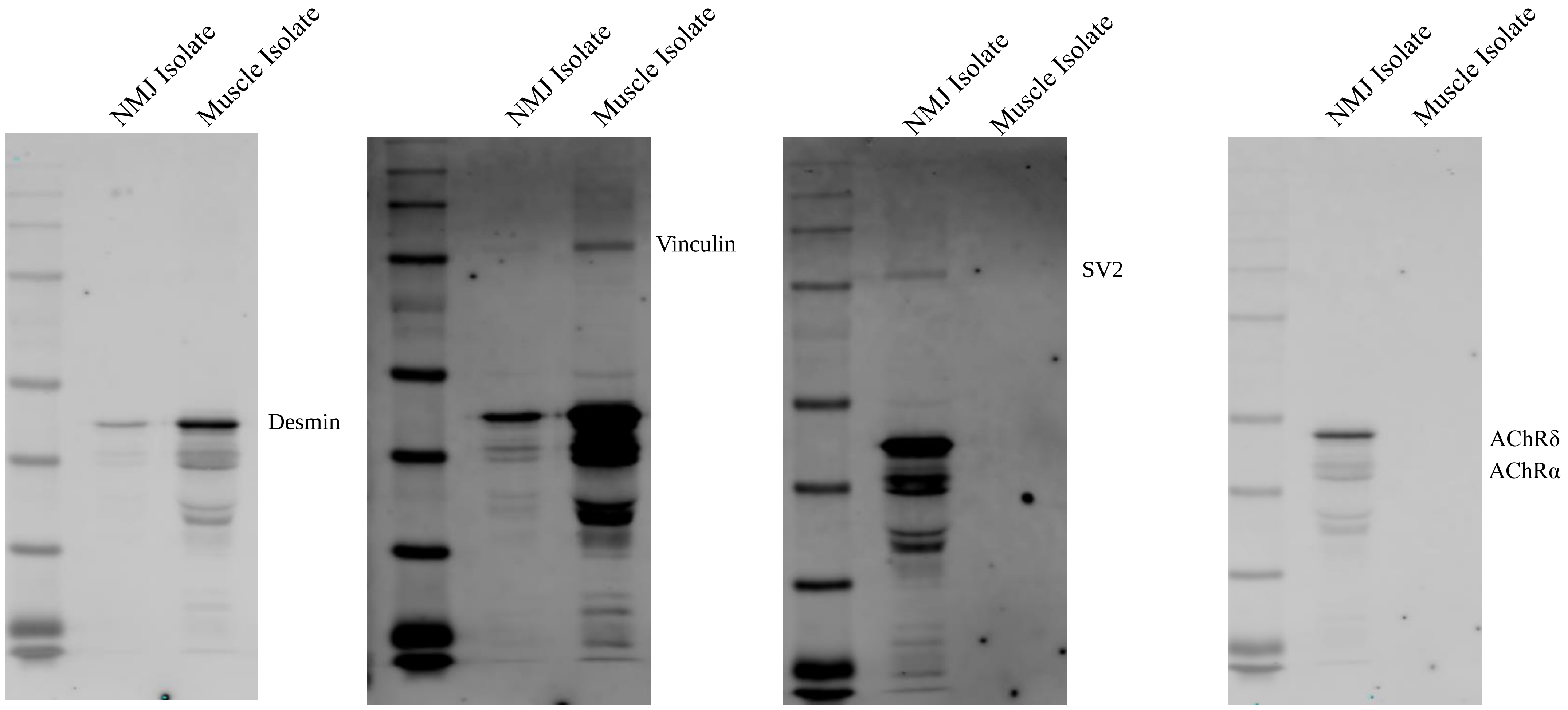

Supplementary 7A

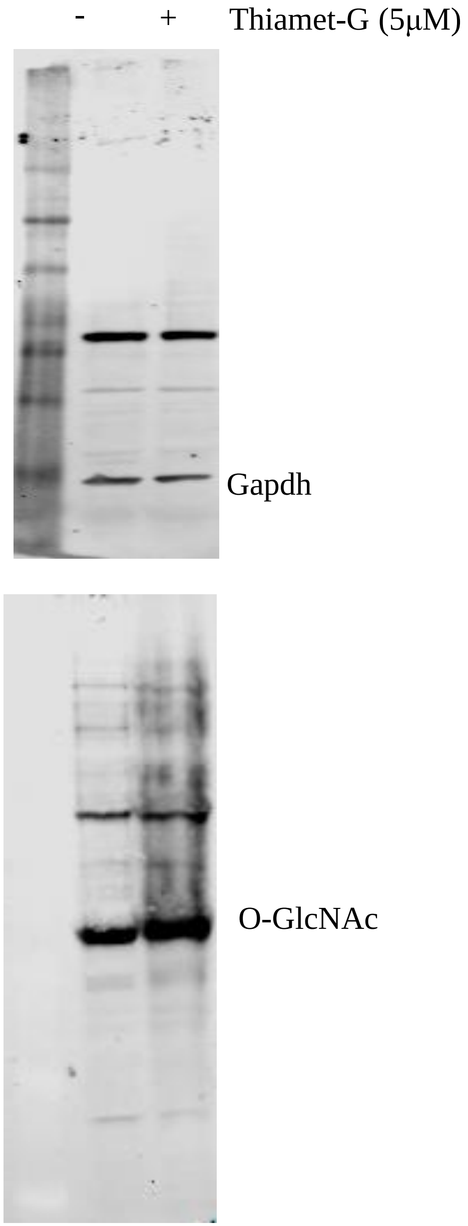

Supplementary 7D

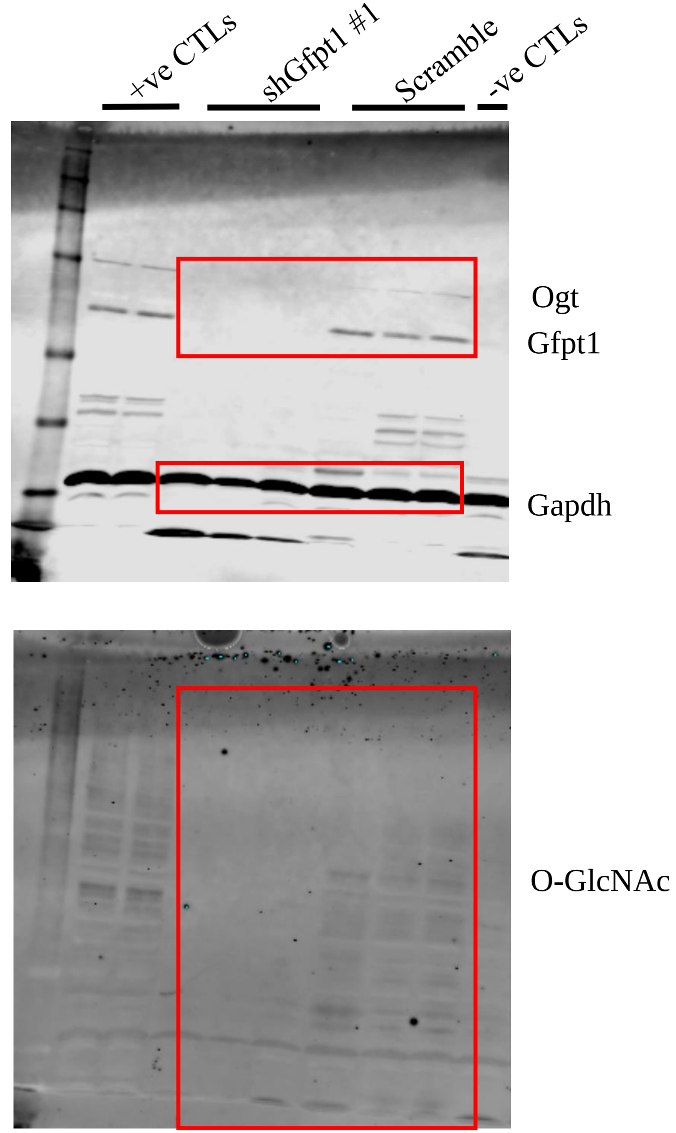

Supplementary 7G

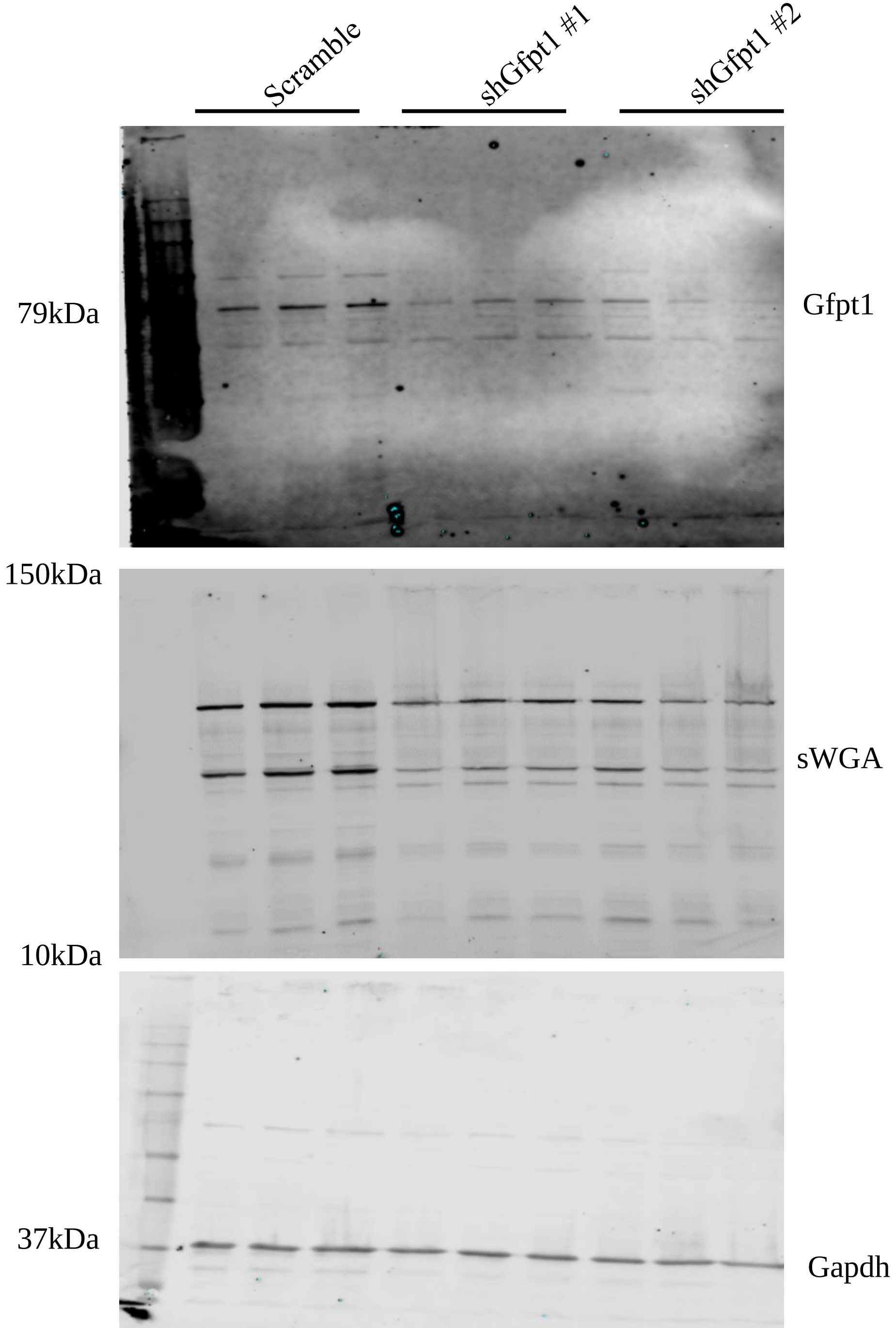

Supplementary 7I

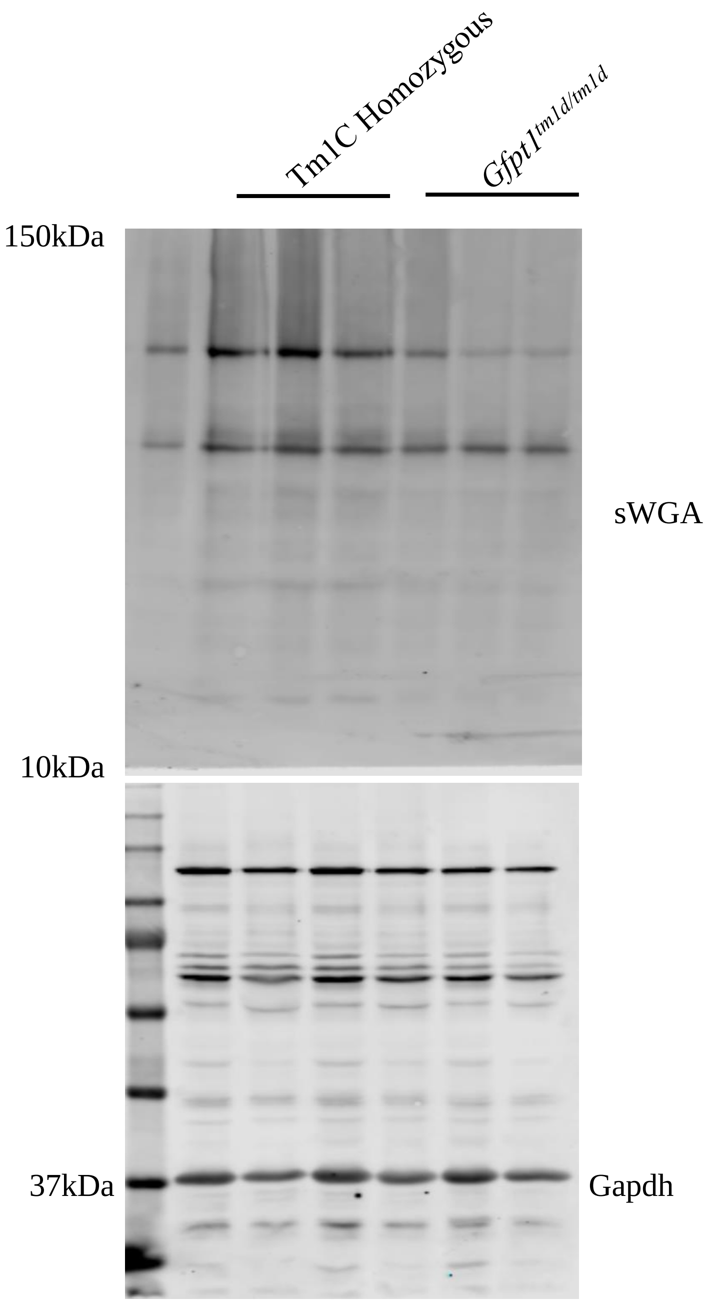

Supplement: Supplementary file 1 [file biomolecules-14-01252-s001.zip › Western blot files_Holland et al 2024_Biomolecules.pdf]
